# The complete mitochondrial genome and description of a new cryptic species of *Benedenia* Diesing, 1858 (Monogenea: Capsalidae), a major pathogen infecting the yellowtail kingfish *Seriola lalandi* Valenciennes in the South-East Pacific

**DOI:** 10.1186/s13071-019-3711-5

**Published:** 2019-10-17

**Authors:** J. Antonio Baeza, Fabiola A. Sepúlveda, M. Teresa González

**Affiliations:** 1 Department of Biological Sciences, Clemson University, 132 Long Hall, Clemson, SC 29634 USA; 2 Smithsonian Marine Station at Fort Pierce, 701 Seaway Drive, Fort Pierce, Florida 34949 USA; 3 Departamento de Biología Marina, Facultad de Ciencias del Mar, Universidad Católica del Norte, Larrondo 1281, Coquimbo, Chile; 4 Laboratorio Eco-parasitologia y Epidemiologia Marina (LEPyEM), Instituto de Ciencias Naturales Alexander von Humboldt, Facultad de Ciencias del Mar y Recursos Biologicos, Universidad de Antofagasta, Angamos 601, Antofagasta, Chile

**Keywords:** Disease, Vector, Marine, Flatworm, Fluke, Purifying selection

## Abstract

**Background:**

The monogenean *Benedenia seriolae* parasitizes fishes belonging to the genus *Seriola*, represents a species complex, and causes substantial impact on fish welfare in aquaculture systems worldwide. This study reports, for the first time, the complete mitochondrial genome of *B. humboldti* n. sp., a new cryptic species from the South-East Pacific (SEP).

**Methods:**

The mitogenome of *B. humboldti* n. sp. was assembled from short Illumina 150 bp pair-end reads. The phylogenetic position of *B. humboldti* n. sp. among other closely related congeneric and confamiliar capsalids was examined using mitochondrial protein-coding genes (PCGs). Morphology of *B. humboldti* n. sp. was examined based on fixed and stained specimens.

**Results:**

The AT-rich mitochondrial genome of *B. humboldti* is 13,455 bp in length and comprises 12 PCGs (*atp*8 was absent as in other monogenean genomes), 2 ribosomal RNA genes, and 22 transfer RNA genes. All protein-coding, ribosomal RNA, and transfer RNA genes are encoded on the H-strand. The gene order observed in the mitochondrial genome of *B. humboldti* n. sp. was identical to that of *B. seriolae* from Japan but different from that of *B. seriolae* from Australia. The genetic distance between *B. humboldti* n. sp. and *B. seriolae* from Japan was high. Minor but reliable differences in the shape of the penis were observed between *Benedenia humboldti* n. sp. and congeneric species.

**Conclusions:**

Phylogenetic analyses based on PCGs in association with differences in the shape of the penis permitted us to conclude that the material from the South-East Pacific represents a new species of *Benedenia* infecting *S. lalandi* off the coast of Chile. The discovery of this parasite represents the first step to improving our understanding of infestation dynamics and to develop control strategies for this pathogen infecting the farmed yellowtail kingfish, *Seriola lalandi*, in the South-East Pacific.
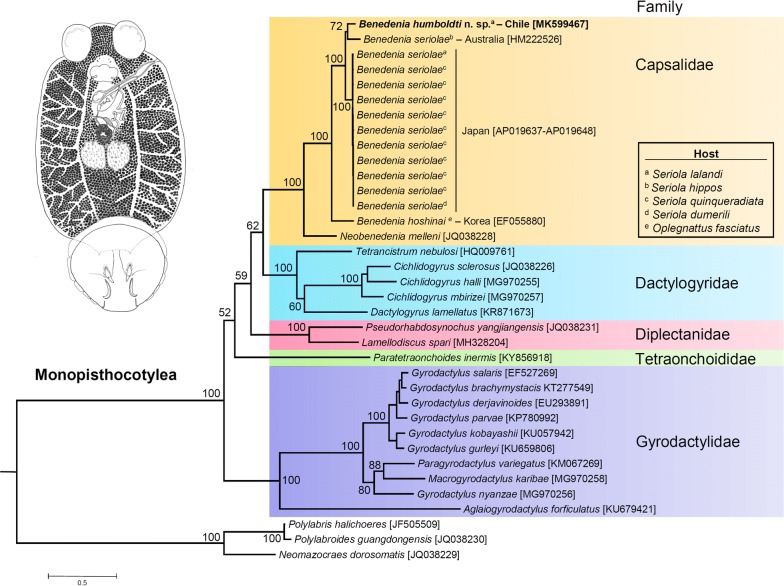

## Background

Monogeneans are a clade of hermaphroditic ectoparasitic flatworms mostly restricted to the skin, fins or gills of marine and freshwater fishes [1]. Monogeneans exhibit direct development and do not require an intermediate host to complete their life-cycle, in contrast to that reported for other parasitic flatworms (i.e. digeneans [1]). Monogenean infestations in farmed fish can and do become pathogenic and outbreaks often result in substantial health issues to the fish population in aquaculture systems worldwide [2, 3]. Some implications of heavy monogenean infestations include direct fish stock loss, depressed fish growth, poor fish health and welfare, reduced value of the market product, and costs associated with monitoring and treatment programs [4].

Among capsalid monogeneans (family Capsalidae Baird, 1853), *Benedenia* spp. attach to fish *via* a pair of anterior attachment organs and an opisthaptor which pierce the epidermis and penetrate the dermis of the host [5]. The presence of large numbers of *Benedenia* spp. parasites during outbreaks causes considerable skin irritation to fish and results in the fish ‘rubbing’ themselves along the bottoms and sides of tanks/cages. Furthermore, *Benedenia* spp. cause skin injuries in fish that often lead to secondary infections by opportunistic pathogens such as bacteria and/or fungi [5]. Unfortunately, genomic resources are limited in monogenean parasites and this poor knowledge is constraining our understanding of infection mechanisms, virulence and pharmacological resistance, among others, in this and other groups of ectoparasites (but there are exceptions [6, 7]).

*Benedenia seriolae* (Yamaguti, 1934) Meserve, 1938, is a particularly persistent problem and a major barrier to efficient finfish production and industry growth worldwide [4, 8]. *Benedenia seriolae* is a well-known parasite on the epidermis of the yellowtail *Seriola quinqueradiata* Temminck & Schlegel, and *S. dumerili* (Risso), cultured in Japan [9] and the kingfish *S. lalandi* Valenciennes, in Australia [3, 10], New Zealand [11], Mexico [12] and Chile [8]. The wide distribution of this parasite might be a consequence of the pan-Pacific distribution of the host species or alternatively, might indicate the existence of a species complex [8]. Using a barcoding approach, it has recently been demonstrated that *Seriola lalandi* in the South-East Pacific (SEP) is parasitized by an entity genetically different from *B. seriolae* in the South-West Pacific (SWP) [8]. Importantly, no major morphological differences were observed among *B. seriolae* parasitizing *S. lalandi* from the SEP and SWP, *S. quinqueradiata* and *S. hippos* Günther. A single trait, however, i.e. the shape of the penis, appears to be dissimilar among *B. seriolae* from different host species and localities (SEP *vs* SWP) [8]. Despite the ecological and aquaculture/fishery importance of *B. seriolae*, no genomic resources exist for this species that could improve our understanding of its life-cycle and its impact on the health of its host populations.

The aim of this study was to describe the complete mitochondrial genome of *B. seriolae* of Sepúlveda & González [8] from the SEP and compare it to previously published mitogenomes of *B. seriolae* from the SWP (Australia and Japan). Importantly, mitochondrial sequence comparison allowed for the description of a new pathogen species, *Benedenia humboldti* n. sp. that infects *S. lalandi* in the SEP. This paper describes the mitochondrial genome of *B. humboldti* n. sp. from the SEP focusing on codon usage profiles and nucleotide composition of protein-coding genes (PCGs). Additionally, the secondary structure of each identified tRNA gene is described and non-coding regions are examined in more detail. Selective constraints in PCGs, including those commonly used for population genetic inference, were explored.

## Methods

### Field collection and sequencing

A total of 4 individuals of *Benedenia humboldti* n. sp. (syn. *Benedenia seriolae* of Sepúlveda & González [8]) were collected with forceps from the skin of the yellowtail kingfish *Seriola lalandi* previously captured by artisanal fishermen in Antofagasta, Chile (23°37′S, 70°24′W). The specimens were immediately fixed in 99% ethanol within a 5 ml centrifuge tube and transported to AUSTRAL-Omics (Valdivia, Chile).

Total genomic DNA was extracted from whole individuals using a High Pure PCR Template Preparation Kit (Roche, Penzberg, Germany), following the manufacturer’s protocol. DNA concentration and purity were assessed using a Quant-iT™ PicoGreen^®^ dsDNA Assay Kit (Thermo Fisher Scientific, Waltham, USA) on a DQ300 Hoefer Fluorometer (Hoefer Inc., Holliston, MA, USA). An Illumina Nextera XT DNA Sample Prep Kit (Illumina, San Diego, CA, USA) was used for whole genome library construction following the manufacturer’s protocol. Briefly, 1 µg of genomic DNA was randomly sheared *via* nebulization, DNA fragment ends were repaired, 3’ adenylated, and ligated to Illumina adapters. The resulting adapter-ligated libraries were PCR-amplified, Illumina indexes added, and pooled for multiplexed sequencing on an Illumina MiSeq sequencer (Illumina) using a pair-end 250 bp run format.

A total of 4,684,263 reads were generated and made available in FASTQ format by the company. The totality of these reads was used for the mitochondrial genome assembly of *B. humboldti* n. sp. from the SEP.

### Mitochondrial genome assembly of *Benedenia humboldti* n. sp.

Contaminants, low quality sequences (Phred scores < 30), Illumina adapters and sequences with less than 50 bp were removed using the software Trimmomatic [13], leaving 3,380,163 pair-end high quality reads for final mitogenome assembly. The mitogenome was assembled *de novo* using the NOVOPlasty pipeline v.1.2.3 [14]. NOVOPlasty uses a seed-and-extend algorithm that assembles organelle genomes from whole genome sequencing (WGS) data, starting from a related or distant single ‘seed’ sequence and an optional ‘bait’ reference mitochondrial genome [14]. To test the reliability of the assembly, we ran NOVOPlasty using two strategies. First, we used a single fragment of the *cox*1 gene available in GenBank (KC633872) as a seed. Secondly, we used the complete mitochondrial genome of *B. seriolae* (HM222526) as a bait reference mitogenome in addition to the same partial *cox*1 seed. We chose to use the mitochondrial genome of *B. seriolae* from the SWP as a ‘bait’ reference because it is the closely related congeneric species with a published mitochondrial genome available on GenBank [15]. The two runs used a kmer size of 49 following the developer’s suggestions [14].

### Annotation and analysis of the *Benedenia humboldti* n. sp. mitochondrial genome

The newly assembled mitochondrial genome was first annotated in the MITOS web server (http://mitos.bioinf.uni-leipzig.de) [16] using the echinoderm/flatworm genetic code (Translation Table 9). Annotation curation and start + stop codons corrections were performed using MEGA6 [17] and Expasy (https://web.expasy.org/). Genome visualization was conducted with OrganellarGenomeDRAW (http://ogdraw.mpimp-golm.mpg.de/index.shtml) [18]. The open reading frames (ORFs) and codon usage profiles of PCGs were analyzed. Codon usage for each PCG was predicted using the invertebrate echinoderm/flatworm code in the Codon Usage web server (http://www.bioinformatics.org/sms2/codon_usage.html). tRNA genes were identified in the software MITFI [19] as implemented in the MITOS web server and the secondary structure of each tRNA was predicted using the tRNAscan-SE v.2.0 web server (http://trna.ucsc.edu/tRNAscan-SE/) [20]. tRNA secondary structures were visualized in the RNAfold web server (http://rna.tbi.univie.ac.at/cgi-bin/RNAWebSuite/RNAfold.cgi) [21].

A relatively short non-coding region in the mitochondrial genome of *B. humboldti* n. sp. from the SEP was examined in more detail. The number of repeats in the region was investigated with the Tandem Repeat Finder v.4.09 web server (http://tandem.bu.edu/trf/trf.html) [22]. We also attempted to discover DNA motifs in this intergenic region using the default options in MEME [23]. Mfold (http://unafold.rna.albany.edu/) and Quickfold (http://unafold.rna.albany.edu/?q=DINAMelt/Quickfold) web servers were used to predict the secondary structure of this region with particular attention to the presence of stem-loops.

Selective constraints in PCGs were explored. Overall values of K_A_ (the number of non-synonymous substitutions per non-synonymous site: K_A_ = d_N_ = S_A_/L_A_), K_S_ (number of synonymous substitutions per synonymous site: K_S_ = d_S_ = S_S_/L_S_) and ω (the ratio K_A_/K_S_) were estimated for each PCG in the software KaKs_calculator v.2.0 [24]. The above values were based on a pairwise comparison between *B. humboldti* n. sp. and *B. seriolae* from Australia (GenBank: HM222526). Next, to identify positively selected sites along the length of each examined sequence, the values of K_A_, Ks and ω were also calculated while adopting a sliding window (window length = 57, step length = 12) that ‘slipped’ along each sequence. The γ-MYN model [25] was used during calculations to account for variable mutation rates across sequence sites [24]. If PCGs are under no selection, selective constraint (purifying selection) or diversifying selection, the ratio ω (= K_A_/K_S_) is expected to be equal to 1, < 1 or > 1, respectively [24].

The phylogenetic position of *B. humboldti* n. sp. and *B. seriolae* from the SWP (Australia and Japan, see below) among other species belonging to the subclass Monopisthocotylea of monogenetic flukes (class Monogenea) was examined. The newly assembled and annotated mitochondrial genome of *B. humboldti* n. sp., 12 sequences for *B. seriolae* from the SWP (available on GenBank), and those of a total of 23 other species of monopisthocotylean flukes retrieved from the GenBank database were used for the phylogenetic analysis conducted using the MitoPhAST pipeline [26]. We used three species of monogeneans in the subclass Polyopisthocotylea as the outgroups for the analysis. MitoPhAST extracts all PCG nucleotide sequences from species available on GenBank and others provided by the user (i.e. *B. humboldti* n. sp. from the SEP), translates each PCG nucleotide sequence to amino acids, conducts alignments for each PCG amino acid sequence using Clustal Omega [27], removes poorly aligned regions with trimAl [28], partitions the dataset and selects best fitting models of sequence evolution for each PCG with ProtTest [29], and uses the concatenated and partitioned PCG amino acid alignments to perform a maximum likelihood phylogenetic analysis in the software RaxML [30]. The robustness of the ML tree topology was assessed by bootstrap reiterations of the observed data 100 times.

### Morphological data

Specimens of *B. humboldti* n. sp. were carefully removed from the skin of freshly sacrificed *S. lalandi* specimens. Nine specimens were fixed and stored in 70% ethanol. Fixed specimens were stained with Ehrlich’s haematoxylin for 15 min and then unstained in 1% HCl diluted in 70% ethanol. Next, each specimen was dehydrated in an ethanol series (70% × 10 min, 80% × 10 min, 90% × 10 min, 95% × 15 min and 100% × 15 min), cleared with xilene and mounted on slides in Canada balsam. Each specimen was examined and compared with other species of *Benedenia* based on morphological characteristics following criteria provided by the specialized literature [31–34]. The specimens were examined under an Olympus BX41 light microscope (Olympus, Tokyo, Japan) connected to a Micrometrics camera (590CU, ACCU-SCOPE Inc., Commack, NY, USA). Images were processed with Micrometric SE Premium software (ACCU-SCOPE Inc., Commack, NY, USA). Body measurements, including total body length and width, haptor length and width, hook length, testes and germarium length and width, pharynx length and width, and penis length are given in micrometers as the range followed by the mean and the number of specimens measured in parentheses. Additionally, 10 live specimens obtained from cultured *S. lalandi* during January 2019 were observed under a stereomicroscope (Olympus SZX7).

## Results and discussion

**Fig. 1:**
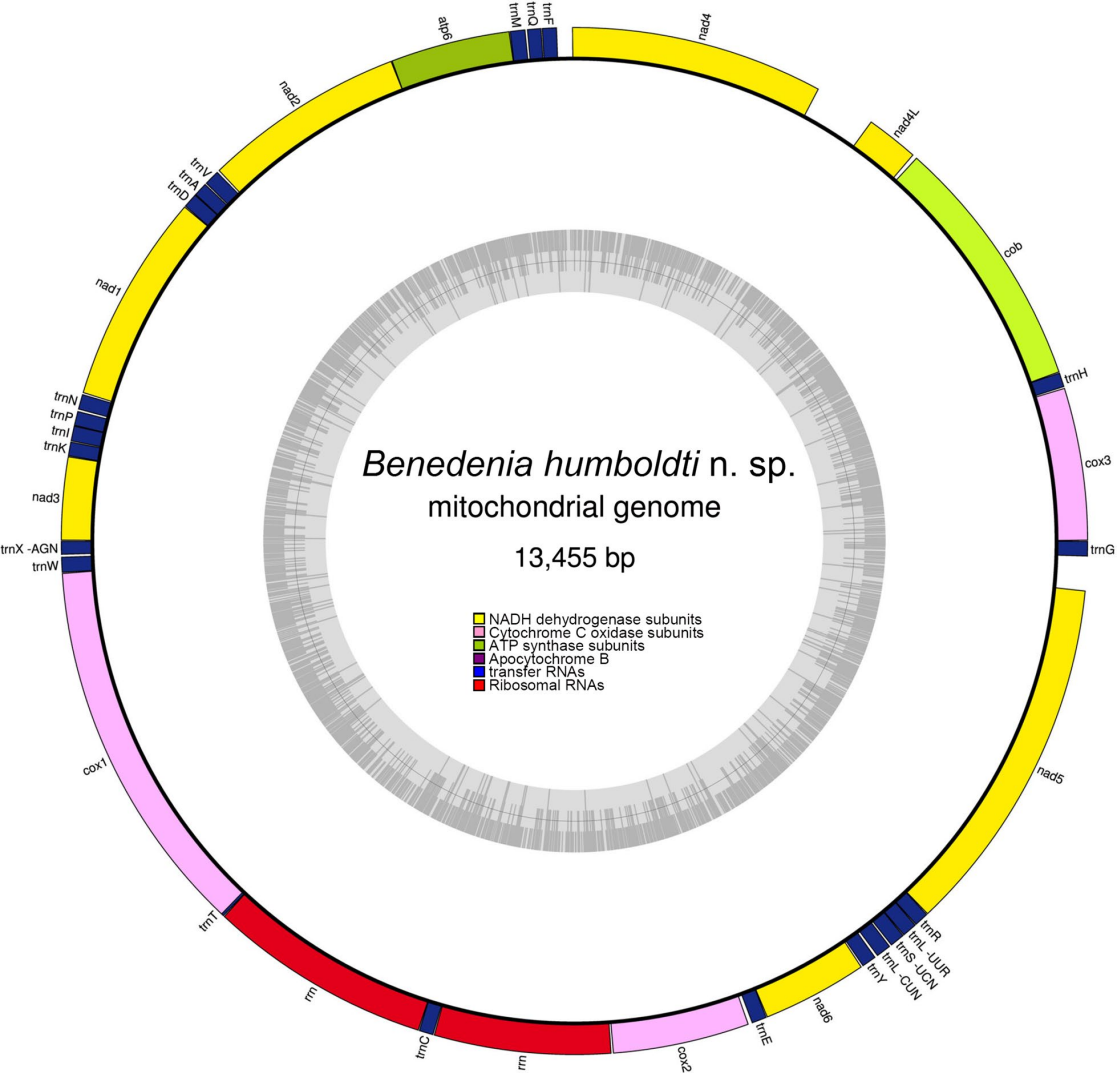
Circular genome map of *Benedenia humboldti* n. sp. mitochondrial DNA. The map is annotated and depicts 12 protein-coding genes (PCGs), 2 ribosomal RNA genes [*rrnS* (*12S* ribosomal RNA) and *rrnL* (*16S* ribosomal RNA)] and 22 transfer RNA (tRNA) genes. The inner circle depicts GC content along the genome. The putative non-coding region likely involved in the initiation of the mitogenome replication is not annotated

The two strategies employed to assemble the mitochondrial genome of *B. humboldti* n. sp. from the SEP in NOVOPlasty resulted in identical sequences. The complete mitochondrial genome of *B. humboldti* n. sp. (GenBank: MK599467) is 13,455 bp in length and comprises 12 protein-coding genes (PCGs), 2 ribosomal RNA genes [*rrnS* (*12S* ribosomal RNA) and *rrnL* (*16S* ribosomal RNA)] and 22 transfer RNA (tRNA) genes. The PCG *atp*8 is lacking in the mitochondrial genome of *B. humboldti* n. sp., in agreement with that reported for the remaining monogeneans whose mitochondrial genomes have been assembled and annotated [15]. All of the PCGs, tRNA genes and the 2 ribosomal RNA genes were encoded on the H-strand (Fig. 1, Table 1).

**Table 1:** Mitochondrial genome of *Benedenia humboldti* n. sp. from the SEP. Arrangement and annotation

Name	Type	Start	Stop	Length (bp)	Start	Stop	Anticodon	Intergenic space
*cox*3	Coding	1	648	648	ATG	TAG		3
trnH(cac)	tRNA	652	715	64			GTG	0
*cob*	Coding	716	1804	1089	ATG	TAG		14
*nad*4*l*	Coding	1819	2046	228	ATG	TAA		0
CR^putative^		2047	2300	254				0
*nad*4	Coding	2301	3373	1071	ATG	TAA		63
*trnF*(ttc)	tRNA	3437	3500	64			GAA	1
*trnQ*(caa)	tRNA	3502	3562	61			TTG	6
*trnM*(atg)	tRNA	3569	3634	66			CAT	0
*atp*6	Coding	3635	4144	510	ATG	TAG		0
*nad*2	Coding	4144	5004	861	GTG	TAG		6
*trnV*(gta)	tRNA	5011	5079	69			TAC	2
*trnA*(gca)	tRNA	5082	5143	62			TGC	0
*trnD*(gac)	tRNA	5143	5208	66			GTC	0
*nad*1	Coding	5209	6102	894	ATG	TAG		4
*trnN*(aac)	tRNA	6107	6173	67			GTT	6
*trnP*(cca)	tRNA	6180	6243	64			TGG	0
*trnI*(atc)	tRNA	6243	6309	67			GAT	1
*trnK*(aag)	tRNA	6311	6372	62			CTT	0
*nad*3	Coding	6373	6726	354	ATG	TAA		1
*trnS*1(agc)	tRNA	6728	6785	58			GCT	8
*trnW*(tga)	tRNA	6794	6859	66			TCA	0
*cox*1	Coding	6860	8470	1611	ATG	TAG		0
*trnT*(aca)	tRNA	8415	8480	66			TGT	0
*rrnL*	rRNA	8481	9431	951				0
*trnC*(tgc)	tRNA	9432	9495	64			GCA	1
*rrnS*	rRNA	9497	10246	750				5
*cox*2	Coding	10,252	10833	582	ATG	TAA		13
*trnE*(gaa)	tRNA	10,847	10913	67			TTC	0
*nad*6	Coding	10,914	11363	450	ATG	TAA		4
*trnY*(tac)	tRNA	11,368	11433	66			GTA	8
*trnL*1(cta)	tRNA	11,442	11509	68			TAG	7
*trnS*2(tca)	tRNA	11,515	11581	67			TGA	0
*trnL*2(tta)	tRNA	11,582	11648	67			TAA	0
*trnR*(cga)	tRNA	11,649	11713	65			TCG	0
*nad*5	Coding	11,714	13249	1536	ATG	TAA		140
*trnG*(gga)	tRNA	13.390	13455	66			TCC	0

**Fig. 2:**
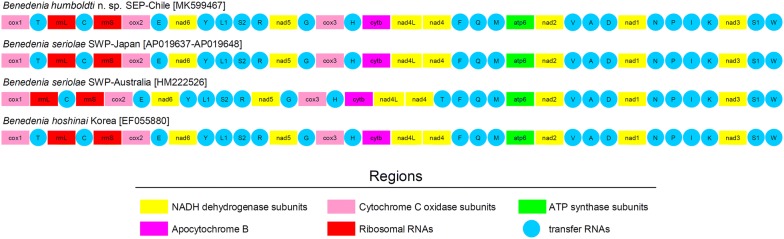
Mitochondrial gene order of *Benedenia humboldti* n. sp. and closely related congeneric species

The gene order observed in *B. humboldti* n. sp. is identical to that reported in *B. seriolae* from Japan (unpublished sequences retrieved from GenBank) and the congeneric *Benedenia hoshinai* Ogawa, 1984 [35]. In turn, gene order of *B. humboldti* n. sp. is different from that of *B*. *seriolae* from Australia [15]. In *B. seriolae* from Australia, the *trnT* gene occurs between *nad*4 and *trnF* while the same gene is located between *cox*1 and *rrnL* in *B. humboldti* n. sp. from the SEP, *B. seriolae* from Japan and *B. hoshinai* (Fig. 2).

Gene overlaps comprising a total of 47 bp were observed in 4 gene junctions: *atp*6-*nad*2 (overlap of 1 bp), *trnA*(*tgc*)*-trnD*(*gtc*) (1 bp), *trnP*(*tgg*)*-trnI* (*gat*) (1 bp) and *cox*1*-trnT*(*tgt*) (40 bp) (Fig. 1, Table 1). In turn, short intergenic spaces ranging in size between 1 and 140 bp were observed in a total of 19 gene junctions. A single relatively long intergenic space involving 254 bp was assumed to be involved in replication initiation of the mitochondrial genome of *B. humboldti* n. sp. as it was found to contain similar features reported for the D-loop/Control Region of other invertebrates (Fig. 1).

Eleven out of the 12 PCGs in the mitochondrial genome of *B. humboldti* n. sp. exhibited the conventional flatworm/echinoderm mitochondrial start codon ATG (Table 1). *nad*2 exhibited the conventional start codon GTG, also observed in the congeneric *B. seriolae* from the SWP [15] and in *Neobenedenia melleni* (MacCallum, 1927) Yamaguti, 1963 [36]. By contrast to *B. humboldti* n. sp. from the SEP and *B. seriolae* from the SWP, the congeneric *B. hoshinai* features the stop codon TAA [35]. All PCGs ended with a complete and conventional termination codon, either TAG or TAA. No PCG terminated with an incomplete stop codon T, as often observed in other monogenean mitochondrial genomes [15, 35, 36].

The PCGs in the mitochondrial genome of *B. humboldti* n. sp. contained an A + T bias with an overall base composition of A = 25.7%, T = 46.6%, C = 11.1% and G = 16.6%. This A + T bias is within the known range reported for mitochondrial genomes in monogenetic flukes and other flatworms and likely affects codon usage. In the PCGs of *B. humboldti* n. sp., the most frequently used codons were UUU (Phe, *n* = 354 times used, 10.95% of the total), UUA (Leu, *n* = 311, 9.62%), AUU (Ile, *n* = 172, 5.32%) and UAU (Tyr, *n* = 153, 4.73%). Less frequently used codons included GCG (Ala, *n* = 1, 0.03%), CGC (Arg, *n* = 1, 0.03%), CCG (Pro, *n* = 2, 0.06%), UCG (Ser, *n* = 4, 0.12%) and ACG (Thr, *n* = 5, 0.16%) (Table 2).

**Table 2:** Codon usage analysis of PCGs in the mitochondrial genome of *Benedenia humboldti* n. sp. from the South-East Pacific

AA	Codon	*n*	Frequency/1000	Fraction	AA	Codon	*N*	Frequency/1000	Fraction
Ala	GCG	1	0.31	0.01	Pro	CCG	2	0.62	0.02
	GCA	12	3.71	0.13		CCA	8	2.47	0.10
	GCT	73	22.57	0.78		CCT	53	16.39	0.65
	GCC	8	2.47	0.09		CCC	18	5.57	0.22
Cys	TGT	78	24.12	0.86	Gln	CAG	12	3.71	0.33
	TGC	13	4.02	0.14		CAA	24	7.42	0.67
Asp	GAT	43	13.30	0.80	Arg	CGG	9	2.78	0.19
	GAC	11	3.40	0.20		CGA	9	2.78	0.19
Glu	GAG	27	8.35	0.44		CGT	28	8.66	0.60
	GAA	35	10.82	0.56		CGC	1	0.31	0.02
Phe	TTT	354	109.46	0.93	Ser	AGG	21	6.49	0.06
	TTC	26	8.04	0.07		AGA	41	12.68	0.12
Gly	GGG	28	8.66	0.16		AGT	103	31.85	0.30
	GGA	20	6.18	0.12		AGC	14	4.33	0.04
	GGT	110	34.01	0.64		TCG	4	1.24	0.01
	GGC	14	4.33	0.08		TCA	18	5.57	0.05
His	CAT	40	12.37	0.69		TCT	125	38.65	0.37
	CAC	18	5.57	0.31		TCC	16	4.95	0.05
Ile	ATA	124	38.34	0.39	Thr	ACG	5	1.55	0.04
	ATT	172	53.18	0.54		ACA	25	7.73	0.21
	ATC	20	6.18	0.06		ACT	78	24.12	0.65
Lys	AAG	51	15.77	1.00		ACC	12	3.71	0.10
Leu	TTG	79	24.43	0.15	Val	GTG	49	15.15	0.20
	TTA	311	96.17	0.58		GTA	82	25.36	0.33
	CTG	19	5.88	0.04		GTT	111	34.32	0.44
	CTA	42	12.99	0.08		GTC	8	2.47	0.03
	CTT	67	20.72	0.13	Trp	TGG	35	10.82	0.49
	CTC	14	4.33	0.03		TGA	36	11.13	0.51
Met	ATG	65	20.10	1.00	Tyr	TAT	153	47.31	0.79
Asn	AAA	92	28.45	0.45		TAC	41	12.68	0.21
	AAT	94	29.07	0.46	Stop	TAG	6	1.86	0.50
	AAC	20	6.18	0.10		TAA	6	1.86	0.50

**Fig. 3:**
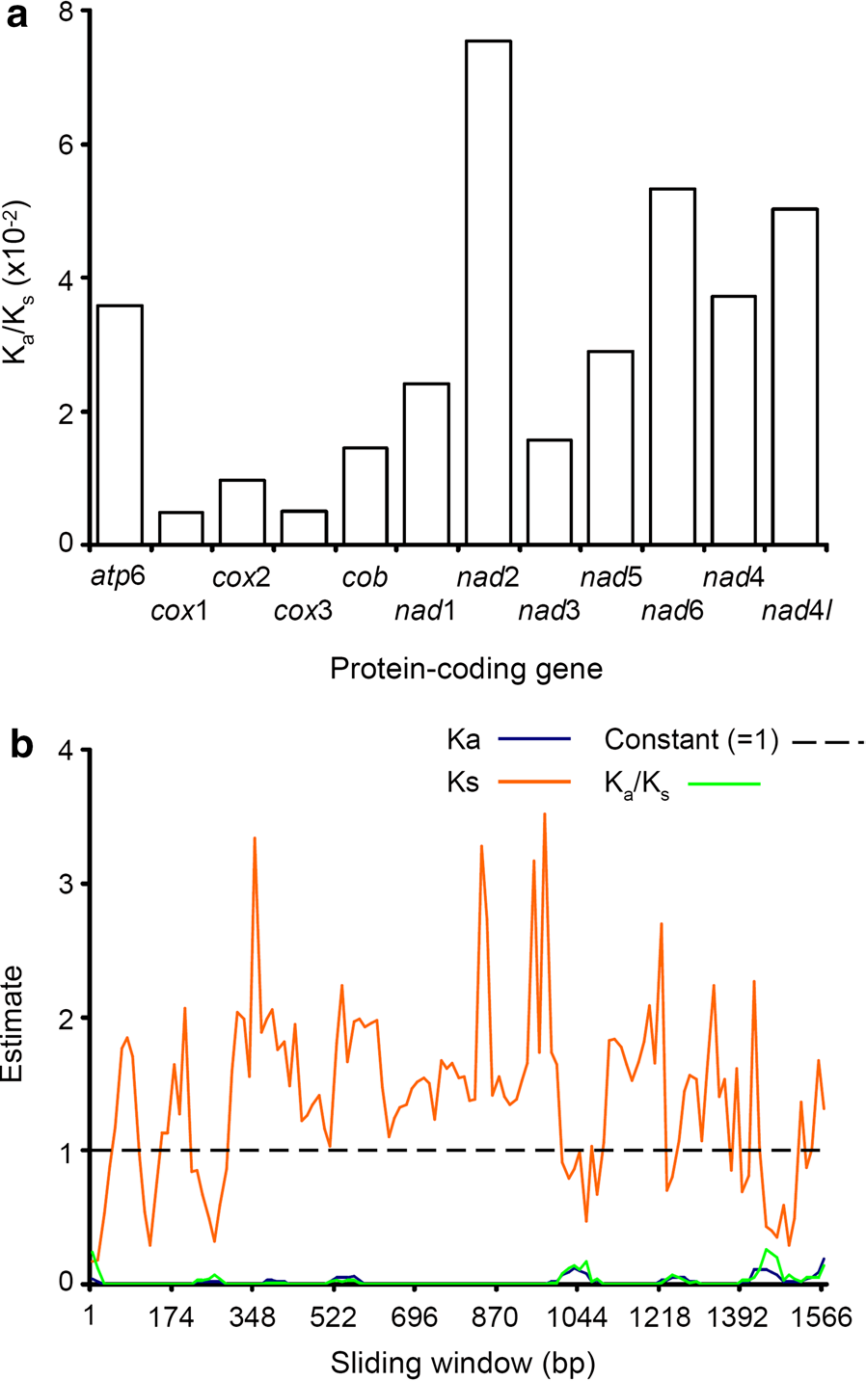
**a** Selective pressure analysis in protein-coding genes present in the mitochondrial genome of *Benedenia humboldti* n. sp. K_A_, K_S_ and K_A_/K_S_ values were calculated using the γ-MYN model. **b** Selective pressure analysis in the *cox*1 gene of *Benedenia humboldti* n. sp. K_A_, K_S_ and K_A_/K_S_ values were calculated using the γ-MYN model and adopting a sliding window of length = 57 and step length = 12. See “Methods” and “Results and discussion” for further details

The K_A_/K_S_ ratios in all mitochondrial PCGs of *B. humboldti* n. sp. showed values < 1, indicating that all these PCGs are evolving under purifying selection. Examination of K_A_/K_S_ ratio values in sliding windows across the length of each PCG sequence further indicated that purifying selection is acting along the entire PCG (Fig. 3, Additional file 1: Table S1). Interestingly, the overall K_A_/K_S_ ratios observed for *cox*1, *cox*2 and *cox*3 (K_A_/K_S_ < 0.00492, 0.00492 and 0.00502, respectively) were an order of magnitude lower than those observed for the remaining PCGs (range: 0.01454–0.07535) suggesting strong purifying selection and evolutionary constraints in the former genes (Fig. 3). Selective pressure in mitochondrial PCG has been poorly studied in monogenetic flukes but a similar pattern of widespread purifying selection in mitochondrial PCGs has been observed in other (marine) invertebrates, including flatworms [15].

**Fig. 4:**
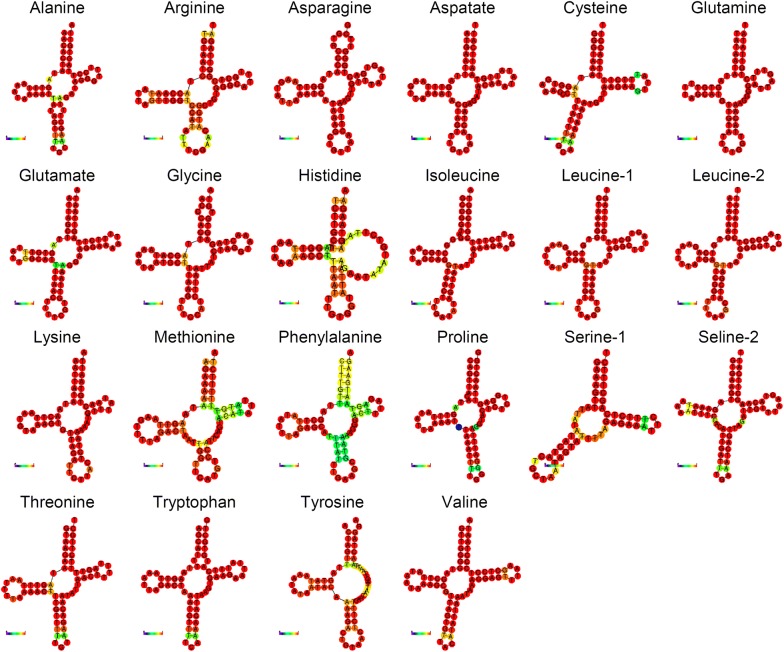
Secondary structure of tRNAs in the mitochondrial genome of *Benedenia humboldti* n. sp. predicted by MITFI and tRNAscan-SE v.2.0

tRNA genes encoded in the mitochondrial genome of *B. humboldti* n. sp. ranged in length from 58 to 61 bp and all but one [*trnS1*(*gct*)] exhibited a standard ‘cloverleaf’ secondary structure as predicted by MITFI. For the *trnS1*(*gct*) gene, MITFI predicted a secondary structure with a missing dihydrouridine arm, a feature also observed in the mitochondrial genomes of *B. seriolae* from the SWP and the closely related *B. hoshinai* and *Neobenedenia melleni* [15, 35, 36]. Interestingly, the RNAfold web server was not able to enforce the secondary structure of the *trnH*(*gtg*) gene predicted by MITFI resulting in the reconstruction of a tRNA with the pseudouridine stem forming a simple loop (Fig. 4). Additionally, the RNAfold web server was not able to enforce the secondary structure of the *trnY*(*gta*) gene predicted by MITFI resulting in the reconstruction of a tRNA with a missing pseudouridine arm. The anticodon nucleotides of all the tRNA genes are consistent with those found in closely related monogenean mitochondrial genomes [36]. *Benedenia hoshinai* represents an exception as it exhibits the anticodon ACG instead of TCG in the *trnR*(*tcg*) gene [35].

The *rrnL* and *rrnS* genes identified in the mitochondrial genome of *B. humboldti* n. sp. were 951 and 750 nucleotides long, respectively. The *rrnL* gene is located between *trnT*(*tgt*) and *trnC*(*gca*). The *rrnS* gene is located close to the *rrnL*, between *trnC*(*gca*) and *cox*2 (Fig. 1). The two genes were A + T biased. The overall base composition of the *rrnL* gene was A = 31.7%, T = 42.8%, C = 10.0% and G = 15.5%, and that of the *rrnS* gene was A = 34.0%, T = 39.5%, C = 10.4% and G = 16.1%.

In *B. humboldti* n. sp., the relatively short 254 bp long non-coding region is located between the *nad*4*l* and *nad*4 genes (Fig. 1). The region was heavily A + T rich with an overall base composition of A = 34.3%, T = 52.4%, C = 7.5% and G = 5.9%. Visual examination of this non-coding region permitted the discovery of a single mononucleotide cytosine repeat near its 5’ end. The Tandem Repeat Finder web server analysis detected one 19-bp-long sequence (5’-TTA TAT ATT ATT TAA ATT T-3’) repeated twice (starting in position 134 and 182 from the 5’ end) in this region. The above features and several AT tandemly repeated sequences observed are in agreement to that observed in the non-coding region of the congenerics *B. seriolae* from the SWP [15] and *B. hoshinai* [35]. Secondary structure prediction analysis in Mfold and Quickfold (assuming 27 °C) resulted each in three possible folding configurations, with a change in Gibbs free energy (ΔG) ranging from − 22.75 to − 23.44 kcal/mol (Additional file 2: Figure S1). In both Mfold and Quickfold, all three reconstructions featured stem-loop structures interspersed along the length of the region (Additional file 2: Figure S1). Nothing is known about replication of the mitochondrial genome in monogeneans [36]. All the features present in the non-coding region of *B. humboldti* n. sp. have been observed before in the putative mitochondrial genome control region/D-loop of other invertebrates [37–39]. Thus, the observed mononucleotide cytosine repeats, high A + T rich base content, tandemly repeated AT sequences and predicted secondary structure(s) suggest an involvement of this non-coding region in the initiation of replication of the mitochondrial genome of *B. humboldti* n. sp.

**Fig. 5:**
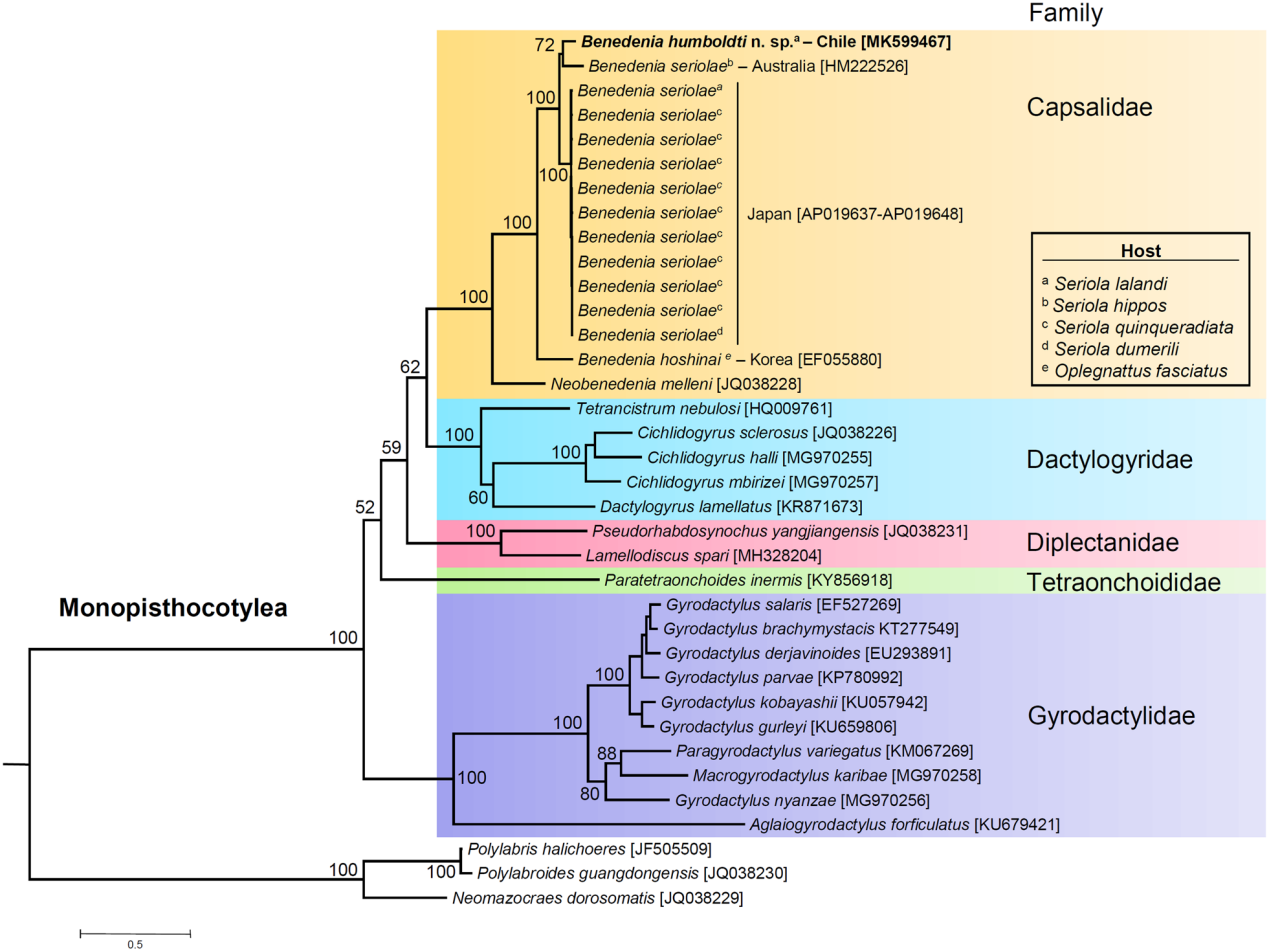
Phylogenetic analysis of *Benedenia humboldti* n. sp. and related species. ‛Total evidenceʼ phylogenetic tree obtained from ML analysis based on a concatenated alignment of amino acids of the protein-coding genes present in the mitochondrial genome of representatives of the subclass Monopisthocotylea. In the analysis, three species of the subclass Polyopisthocotylea were used as the outgroup. Numbers at the branches represent bootstrap values. The optimal molecular evolution model estimated with ProtTest as implemented in NOVOPlasty was the mtZOA+F+I+G4 model; this model was also found to be optimal and applied to two different partitions (partition 1: *atp*6 + *nad*1 + *nad*3 + *nad4l* + *nad*2, partition 2: *cob* + *cox*1 + *cox*2)

The ML phylogenetic tree confirmed the monophyly of the subclass Monopisthocotylea within the class Monogenea and placed *B. humboldti* n. sp. from the SEP in a monophyletic clade together with *B. seriolae* from the SWP (Australia and Japan), *B. hoshinai,* and *Neobenedenia melleni*, in agreement with previous phylogenetic studies using a combination of partial mitochondrial and nuclear genes [8] (Fig. 5). Within this clade, *B. humboldti* n. sp. was sister to *B. seriolae* from Australia, a parasite of *Seriola hippos*. All *B. seriolae* from Japan clustered together into a well-supported monophyletic clade that was sister to the clade comprising *B. humboldti* n. sp. from the SEP and *B. seriolae* from Australia. Additional well-supported clades within the Monopisthocotylea included the families Dactylogiridae, Diplectanidae and Gyrodactylidae. Several nodes located near the root of the phylogenetic tree were well supported (Fig. 5). The above suggests that mitochondrial genomes alone will likely have enough phylogenetic information to reveal relationships at higher taxonomic levels within the subclass Monopisthocotylea.

The gene order herein reported for *B. humboldti* n. sp. is different than that of *B. seriolae* from Australia but identical to that reported for *B. seriolae* from Japan (see above). Importantly, the genetic distance between *B. humboldti* n. sp. and *B. seriolae* from the SWP (both Australia and Japan) was large (p-distance full mitogenome = 0.16; *cox*1 = 0.127; *cytb* = 0.134; *rrnL* = 0.096) and comparable to that previously calculated for other pairs of morphologically dissimilar species of *Benedenia* [8]. Considering the information above, we examined the morphology of our specimens (from the SEP) in more detail and found minor but reliable differences when compared with *B. seriolae* from the SWP. In the following, we describe *B. humboldti* n. sp., a pathogen infecting *S. lalandi* off the coast of Chile.

**Family Capsalidae Baird, 1853**

**Genus** ***Benedenia*** **Diesing, 1858**

***Benedenia humboldti*** **Sepúlveda, González & Baeza, n. sp.**

Syn. *Benedenia seriolae* of Sepúlveda & González [8]

***Type-host***: *Seriola lalandi* Valenciennes (Perciformes: Carangidae).

***Type-locality***: Off Antofagasta (23°37′S, 70°24′W), Chile.

***Other localities***: Off northern coast of Chile, from Antofagasta to Valparaíso (24°S, 70°W to 33°S, 71°W), and Archipelago of Juan Fernández (33°S, 80°W) [8].

***Type-material***: The holotype (stained whole mount) was deposited in the Chilean Museum of Natural History, Santiago, Chile, under the accession number MNHNCL PLAT-15005. Paratypes fixed in ethanol were deposited in the Chilean Museum of Natural History (3 lots: MNHNCL PLAT-15006, MNHNCL PLAT-15007 and MNHNCL PLAT-15008).

***Site on host***: Body surface.

***Prevalence***: 16% (29 out of 180 examined fish).

***ZooBank registration***: To comply with the regulations set out in article 8.5 of the amended 2012 version of the *International Code of Zoological Nomenclature* (ICZN) [40], details of the new species have been submitted to ZooBank. The Life Science Identifier (LSID) of the article is urn:lsid:zoobank.org:pub:367FDE8C-75A7-4A09-A9B4-9E848F20E0F7. The LSID for the new name *Benedenia humboldti* is urn:lsid:zoobank.org:act:D4E5F88F-E1C5-445A-BF69-C3D4AE79CAC2.

***Etymology***: The specific epithet refers to Alexander von Humboldt.

### Description

**Fig. 6:**
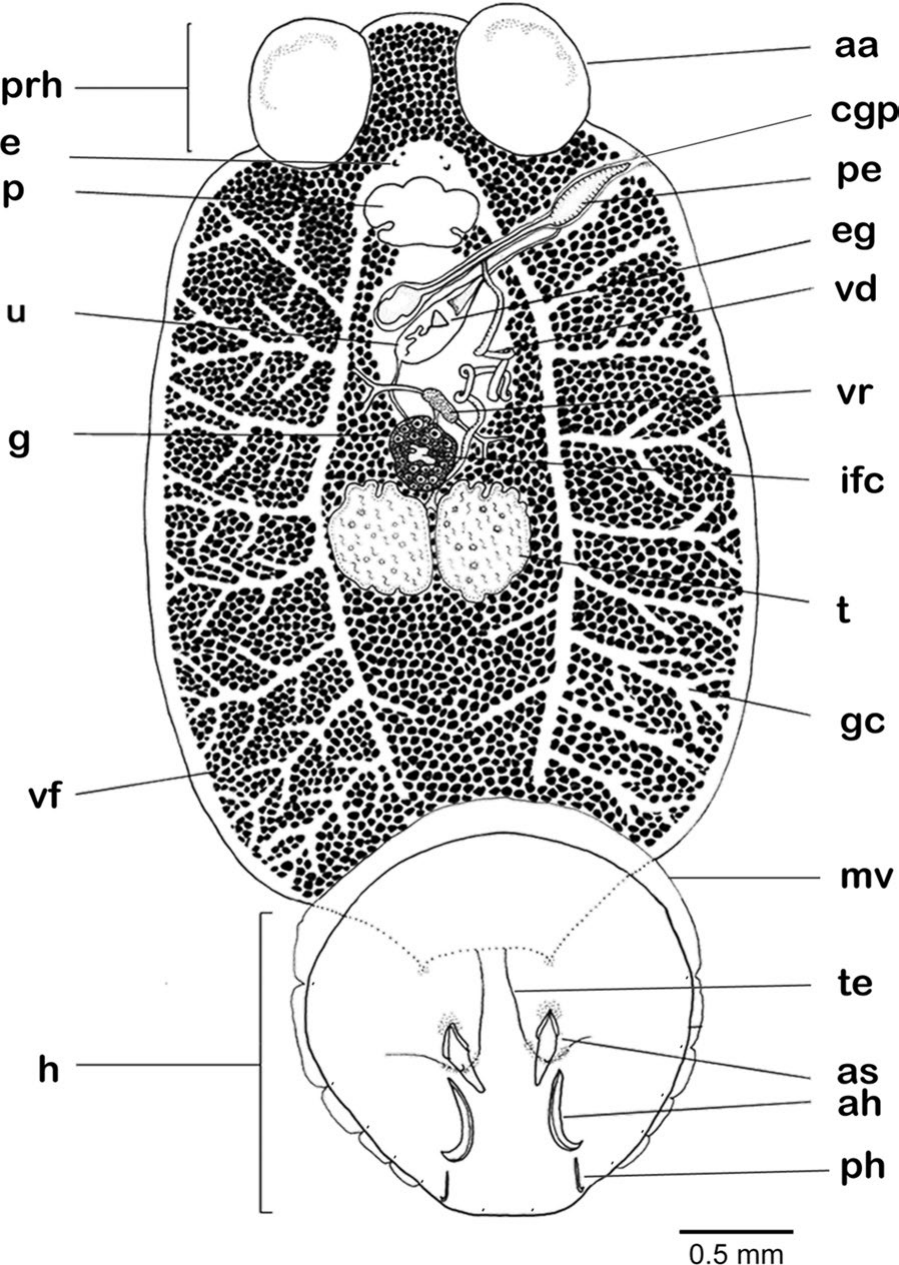
*Benedenia humboldti* n. sp. Entire worm, ventral view (composite drawing mostly from type-specimens). *Abbreviations*: aa, anterior attachment organ; ah, anterior hamulus; as, accessory sclerite; cgp, common genital pore; e, eye-spots; eg, egg; g, germarium; gc, gut caeca; h, haptor; ifc, internal fertilization chamber; mv, marginal valve; p, pharynx; pe, penis; ph, posterior hamulus; prh, prohaptor; t, testis; te, tendons; u, uterus; vd, vas deferens; vf, vitelline follicle; vr, vitelline reservoir

**Fig. 7:**
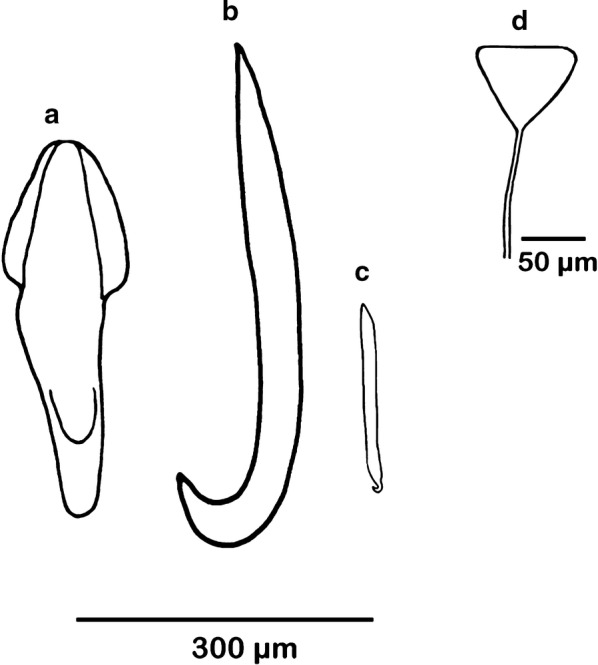
*Benedenia humboldti* n. sp. Haptoral sclerites and egg shape. **a** Accessory sclerite. **b** Anterior hamulus. **c** Posterior hamulus. **d** Egg

**Fig. 8:**
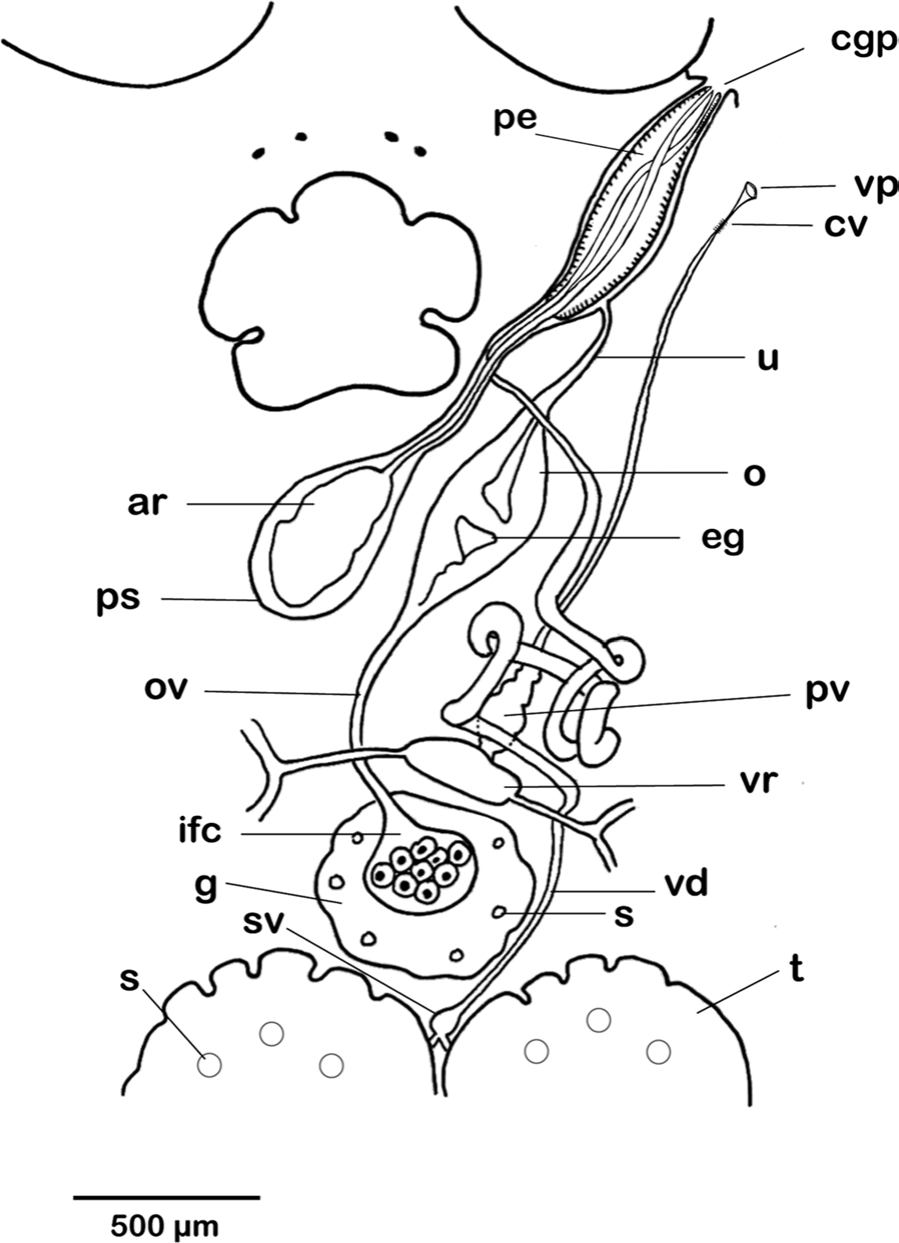
*Benedenia humboldti* n. sp. Reproductive system, ventral view, with vitellarium omitted. *Abbreviations*: ar, accessory gland reservoir; cgp, common genital pore; ifc, internal fertilization chamber; cv, constricted region of vagina; eg, egg; g, germarium; o, oötype; ov, ovovitelline duct; pe, penis; pv, proximal storage region of vagina; s, columnar structure; sv, seminal vesicle; t, testis; u, uterus; vd, vas deferens; vp, vaginal pore; vr, vitelline reservoir

**Fig. 9:**
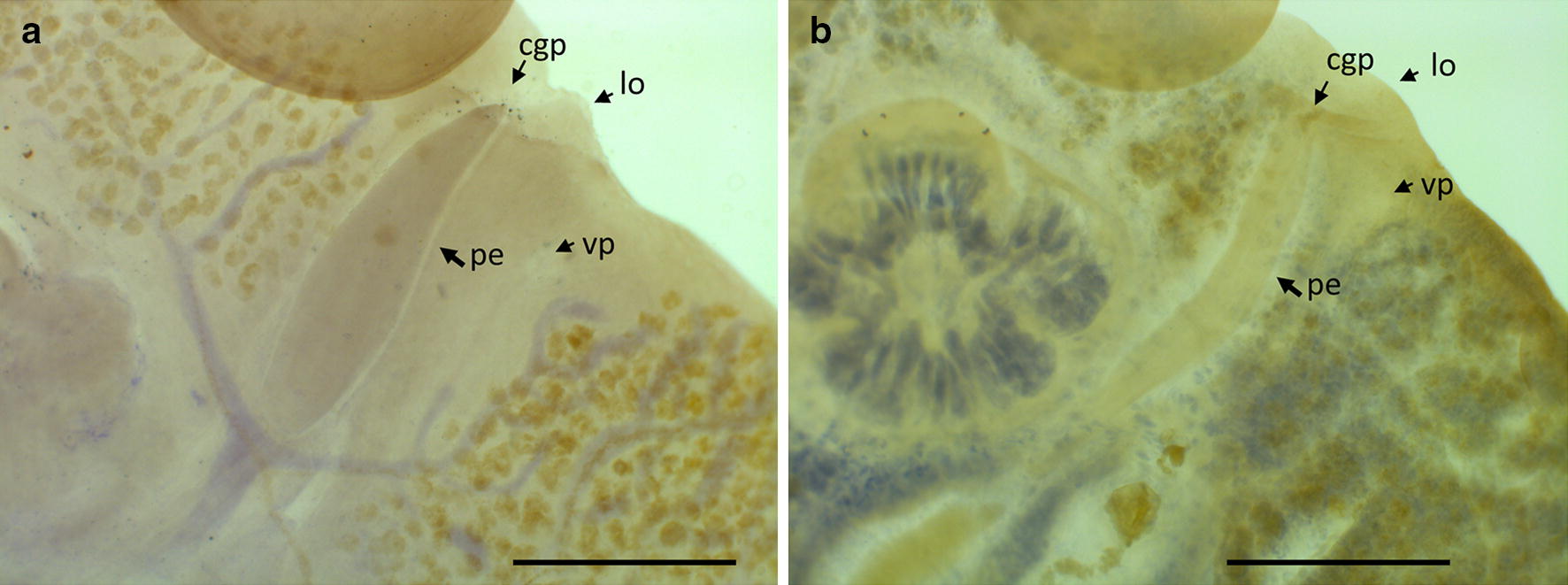
*Benedenia humboldti* n. sp. Penis shape of *Benedenia humboldti* n. sp. from *Seriola lalandi* off Chile (**a**) and *B. seriolae* from *S. quinqueradiata* off Japan (**b**). Specimens of *B. seriolae* were kindly donated by Dr Shirakashi. *Abbreviations*: cgp, common genital pore; lo, lobe; pe, penis; vp, vaginal pore. *Scale-bars*: 500 µm

[Based on 10 live specimens and nine flattened, preserved, stained and mounted adult specimens; Figs. 6, 7, 8, 9]. Total length including haptor 5526–11,210 (8147; *n* = 9); maximum body width at level of testes, 2553–5045 (3791; *n* = 9). Haptor slightly circular, with wider anterior portion, 1537–3289 (2232; *n* = 9) long, 1677–3421 (2376; *n* = 9) wide (Fig. 6). Accessory sclerites 2, located centrally on haptor, 305–654 (430; *n* = 5) long (Figs. 6, 7a). Anterior hamuli 2, elongated, strongly recurved distally, 374–705 (530; *n* = 5) long (Figs. 6, 7b); their proximal ends just overlap with proximal ends of accessory sclerites. Posterior hamuli 2, 83–118 (104; *n* = 4) long (Figs. 6, 7c). Hooklets 14, along haptor periphery. Tendons of extensive body musculature passing through proximal notch of accessory sclerites. Marginal valve present, substantially wider anteriorly (Fig. 6).

Anterior attachment organs 2, approximately circular or elliptical, 600–1237 (934; *n* = 9) long, 593–1070 (797; *n* = 9) wide. Pharynx 343–762 (589; *n* = 9) long, 412–952 (732; *n* = 9) wide. Eye-spots 2 pairs, dorsal, just anterior to pharynx. Gut caeca branched, not united posteriorly (Fig. 6). Testes 2, 777–1485 (1143; *n* = 9) long, 637–1380 (1050; *n* = 9) wide; each testis with variable numbers of columnar structures. Vas deferens widens to form small seminal vesicle at level just posterior to germarium, ascends along left side of germarium, and coils extensively before penetrating lateral wall of penis-sac about halfway along its length (Fig. 8); within penis, vas deferens follows longitudinal path towards distal tip of penis. Accessory gland reservoir prominent, occupies proximal quarter of penis-sac; duct long, joins vas deferens near distal tip of penis. Penis muscular, with size directly proportional to body size, 432–935 (694; *n* = 9) long, with proximal third broader than distal third, protrusible *via* common genital duct and submarginal, dorsolateral, common genital aperture (Figs. 6, 8, 9). Prominent dorsal rounded lobe present on left of common genital aperture (lo, Fig. 9). Long duct connects accessory gland reservoir to penis-sac. Glands of Goto not observed. Germarium globular, compact, 504–1100 (796; *n* = 9) long, 459–1110 (865; *n* = 9) wide, with relatively large internal fertilization chamber from which ovovitelline duct arises up to oӧtype (Fig. 8); columnar structures similar to those in testes present (s, Fig. 8). Vaginal opening on dorsal surface, posterior to common genital aperture. Vaginal opening leads to short straight duct, 83–136 (118; *n* = 3) long, narrowing into constricted region (cv, Fig. 8); vaginal duct travels posteriorly to enlarged proximal storage region of vagina communicating with vitelline reservoir. Oötype with thin-walled proximal region and bulbous thick-walled distal muscular region. Uterus opens into genital atrium at level of penis base. In live but not fixed specimens, connection between vitelline reservoir and oötype was detected during egg formation. Eggs tetrahedral (Fig. 7).

### Remarks

Of the 28 described species of *Benedenia*, *B. humboldti* n. sp. differs from *B. beverleyburtonae* Whittington & Deveney, 2011, *B. acanthopagri* (Hussey, 1986), *B. anticavaginata* Byrnes, 1986, *B. lutjanis* Whittington & Kearn, 1993 and *B. ernsti* Deveney & Whittington, 2010, because the latter five species possess a vaginal opening located anteriorly to the common genital pore [32, 34] or posteriorly to the left testis [33]. In *B. ovata* (Goto, 1894), the vaginal pore opens at mid-body length, between the germarium and the common genital pore, and in *B. sciaenae* (Van Beneden, 1852) Odhner, 1905, male and female pores are separated but very closely located [32].

In contrast to the species listed above, in *B. humboldti* n. sp. the vaginal opening is located close to the left margin of the body and is posterior to the common genital pore like in most species of *Benedenia*. Additionally, *B. humboldti* n. sp. differs from *B. rohdei* Whittington, Kearn & Beverley-Burton, 1994, and *B. jaliscana* Bravo-Hollis, 1951, because the latter two species have the distal tip of penis armed with a sclerite [32, 41]. The specimens of *B. humboldti* n. sp. can be differentiated from other species of *Benedenia* described and/or reported from biogeographical regions other than the South Pacific by a combination of the following characters: body size; position of the median haptoral sclerites; size of haptor relative to body size; shape of the accessory sclerites and hamuli; relation between size of accessory sclerites and anterior hamuli [32–34].

*Benedenia humboldti* n. sp. most closely resembles *B. seriolae* from *Seriola* spp. and *B. hendorffii* (Linstow, 1889) Stiles & Hassall, 1908, from *Coryphaena hippurus* Linnaeus. *Benedenia humboldti* n. sp. and *B. seriolae* parasitize fishes belonging to the genus *Seriola* [32]. The original description of *B. seriolae* [42] from *S. aureovittata* (= *S. lalandi*) was complemented [31, 43] with specimens obtained from *S. quinqueradiata* off Japan. Later, Whittington et al. [32] added morphological and morphometric information for *B. seriolae* from *S. lalandi* collected off Australia and Chile and suggested that *B. seriolae* was a cosmopolitan species infecting a variety of carangid fishes. Nonetheless, molecular analyses demonstrated that the species of *Benedenia* (identified as *B. seriolae*) from *S. lalandi*, *S. quinqueradiata* and *S. hippos* were genetically dissimilar; genetic distance was above 13% among the species but there was no significant morphometric disparity among them [8]. The only morphological attribute that differentiates *B. humboldti* n. sp. (syn. *B. seriolae* of Sepúlveda & González [8]) from the SEP and *B. seriolae* from the SWP is penis shape. *Benedenia humboldti* n. sp. has a pine-nut elongated (lanceolated) penis shape while *B. seriolae* from the SWP has a blunt penis tip (Fig. 9).

*Benedenia hendorffii* was described by von Linstow [44] from the body surface of *Coryphaena hippurus* (L.) off Chile. No type-material was deposited by von Linstow [44] and Price [45] redescribed *B. hendorffii* based on a single specimen from an unknown fish species captured off Spokane, Washington, USA. Whittington et al. [32] checked the material by Price [45] and confirmed, based on this unique specimen, the identity of *B. hendorffii*. A comparison of *B. humboldti* n. sp. with the description and illustrations of *B. hendorffii* by von Linstow [44] revealed important differences between the two species such as the absence of a penis-sac (or a similar muscular organ), the existence of a separated uterine duct extending the length of the penis complex that opens separately and posteriorly to the male pore, and the absence of a vagina in *B. hendorffii*. Additionally, *B. humboldti* n. sp. differs from *B. hendorffii* redescribed by Price [45] by a combination of characters such as the accessory sclerites (striated in *B. hendorffii*) and the curvature of the distal end of the anterior hamulus (more open in *B. humboldti* n. sp. than in *B. hendorffii*). The penis shape of *B. hendorffii* looks similar to that of *B. seriolae*. We suggest that *B. hendorffii* should be considered a *species inquirenda* given the lack of type-material in the original description by von Linstow [44]. In his description, von Linstow commented that the host specimens of *C. hippurus* were captured together with *Seriola* sp. hosts, which raises doubts about the correct identification of the host from which *B. hendorffii* specimens were taken. In addition, there is a lack of information about the host species from which the specimen redescribed as *B. hendorffii* by Price [45] was obtained. Finally, *B. hendorffii* has been found rarely on *C. hippurus*, and the presence of this monogenean in *C. hippurus* has been considered accidental [46].

## Conclusions

In conclusion, this study assembled for the first time the mitochondrial genome of *Benedenia humboldti* n. sp., a cryptic species of great economic interest given its parasitic association with the yellowtail kingfish, *Seriola lalandi*, in aquaculture facilities from the SEP [8, 47, 48]. An integrative approach that included the study of the complete mitochondrial genome of *Benedenia humboldti* n. sp. from the SEP and *B. seriolae* from the SWP plus phylogenetic analyses and interrogation of morphological traits permitted us to confirm the existence of this new cryptic species in the genus *Benedenia*. The correct identity of this parasite represents the first step towards improving our understanding of infestation dynamics and control strategies of this pathogen in farmed *S. lalandi* in the SEP.

## Supplementary information

**Additional file 1: Table S1.** Selective pressure analysis in PCGs of *B. humboldti* n. sp. from the SEP. K_A_, K_S_ and K_A_/K_S_ values were calculated using the γ-MYN model and adopting a sliding window of length = 57 and step length = 12.

**Additional file 2: Figure S1.** Secondary structure prediction analysis of the non-coding putative D-loop/CR in the mitochondrial genome of *B. humboldti* n. sp. from the SEP.

## Data Availability

Data supporting the conclusions of this article are included within the article and its additional files. The mitochondrial genome sequence is available in the GenBank database under the accession number MK599467.

## Abbreviations

K_A_: number of nonsynonymous substitutions per nonsynonymous site
K_S_: number of synonymous substitutions per synonymous site
ML: maximum-likelihood phylogenetic analysis
ORFs: open reading frames
PCGs: protein coding genes
*rrnS*: *12S* ribosomal RNA
*rrnL*: *16S* ribosomal RNA
SEP: South-East Pacific
SWP: South-West Pacific
tRNA: transfer RNA
ω: ratio K_A_/K_S_
ΔG: Gibbs free energy
