# Supplementary material for: The complete mitochondrial genome and description of a new cryptic species of Benedenia Diesing, 1858 (Monogenea: Capsalidae), a major pathogen infecting the yellowtail kingfish Seriola lalandi Valenciennes in the South-East Pacific
Source: Parasit Vectors. 2019 Oct 17;12:490. doi: 10.1186/s13071-019-3711-5 (PMC6798380; doi:10.1186/s13071-019-3711-5)
Supplement: Supplementary file 1 — Additional file 1: Table S1. Selective pressure analysis in PCGs of B. humboldti n. sp. from the SEP. KA, KS and KA/KS values were calculated using the γ-MYN model and adopting a sliding window of length = 57 and step length = 12. [file 13071_2019_3711_MOESM1_ESM.pdf]

The complete mitochondrial genome and description of a new cryptic species of *Benedenia* Diesing, 1858 (Monogenea: Capsalidae), a major pathogen infecting the yellowtail kingfish *Seriola lalandi* Valenciennes, 1833 in the South-East Pacific

J. Antonio Baeza[1, 2, 3,*]

Fabiola Sepulveda[4]

Teresa Gonzalez[4,*]

[1] *Department of Biological Sciences, 132 Long Hall, Clemson University, Clemson, SC 29634, USA.*

[2] *Smithsonian Marine Station at Fort Pierce, 701 Seaway Drive, Fort Pierce, Florida 34949, USA.*

[3] *Departamento de Biología Marina, Facultad de Ciencias del Mar, Universidad Católica del Norte, Larrondo 1281, Coquimbo, Chile.*

[4] *Laboratorio Eco-parasitologia y Epidemiologia Marina (LEPyEM), Instituto de Ciencias Naturales Alexander von Humboldt, Facultad de Ciencias del Mar y Recursos Biologicos, Universidad de Antofagasta, Angamos 601, Antofagasta, Chile.*

Additional file 1: Table S1. Selective pressure analysis in PCGs of *Benedenia humboldti* n. sp. from the South-East Pacific. K_A, K_S and K_A / K_S values were calculated using the γ-MYN model and adopting a sliding window of length = 57 and step length = 12. See methods and results for further details.

Gene/Segment		Method	Ka	Ks	Ka/Ks	P-Value
ATP6						
	1	GMYN	0.043551	0.368935	0.118046	0.083344
	2	GMYN	0.040418	1.06815	0.037839	0.003804
	3	GMYN	0.086593	1.41798	0.061068	NA
	4	GMYN	0.164684	1.5475	0.106419	0
	5	GMYN	0.138325	1.90157	0.072743	5.31E-05
	6	GMYN	0.332032	1.11888	0.296753	0.026149
	7	GMYN	0.214471	2.9515	0.072665	7.84E-05
	8	GMYN	0.208874	1.39229	0.150022	0.000496
	9	GMYN	0.29953	1.70573	0.175603	0.01605

10	GMYN	0.541752	0.681008	0.795515	0.771517
11	GMYN	0.391516	1.10902	0.353028	0.04256
12	GMYN	0.363207	0.909408	0.399388	0.135455
13	GMYN	0.333285	0.621628	0.536149	0.139048
14	GMYN	0.355765	0.237169	1.50005	0.733022
15	GMYN	0.162408	0.558436	0.290827	0.068798
16	GMYN	0.164903	0.985328	0.167358	0.065174
17	GMYN	0.170096	1.34391	0.126568	0.028827
18	GMYN	0.089264	2.16407	0.041248	0.031448
19	GMYN	0.020569	0.655547	0.031376	NA
20	GMYN	1.28E-10	0.547348	2.33E-10	NA
21	GMYN	7.16E-11	1.4067	5.09E-11	NA
22	GMYN	0.020483	1.40382	0.014591	NA
23	GMYN	0.020922	1.59674	0.013103	1.47E-08
24	GMYN	0.022628	1.86342	0.012143	2.28E-07
25	GMYN	0.022603	1.86091	0.012146	2.17E-07
26	GMYN	0.044361	1.12905	0.039291	0.000176
27	GMYN	0.022328	1.35396	0.016491	1.30E-05
28	GMYN	0.044045	1.23423	0.035686	0.000131
29	GMYN	0.065499	1.41947	0.046143	0.001213
30	GMYN	0.090545	1.19828	0.075562	0.003529
31	GMYN	0.204802	1.0458	0.195832	0.025392
32	GMYN	0.200895	1.47015	0.136649	0.003836
33	GMYN	0.205264	1.41379	0.145187	0.020514
34	GMYN	0.176817	1.25355	0.141053	0.00039
35	GMYN	0.080856	1.72352	0.046913	8.99E-06
36	GMYN	2.48E-10	1.79874	1.38E-10	0
37	GMYN	NA	0.772115	NA	0
38	GMYN	1.11E-15	0.69027	1.61E-15	0

Gene/Segment	Method	Ka	Ks	Ka/Ks	P-Value
Cox1					
	1	0.041657	0.170644	0.244114	0.310657
	2	0.020723	0.17842	0.11615	0.200071
	3	NA	0.535852	NA	0
	4	0	0.885091	0	0
	5	1.11E-15	1.16923	9.50E-16	0
	6	1.11E-15	1.76525	6.29E-16	0
	7	0	1.84841	0	0
	8	0	1.70052	0	0
	9	0	0.975787	0	0
	10	1.11E-15	0.542892	2.05E-15	0

11	0	0.293631	0	0
12	0	0.76359	0	0
13	0	1.13192	0	0
14	2.22E-15	1.13557	1.96E-15	0
15	0	1.63922	0	0
16	2.22E-15	1.27	1.75E-15	0
17	1.11E-15	2.06484	5.38E-16	0
18	0	0.843945	0	0
19	0.022531	0.851347	0.026465	0.000137
20	0.021218	0.667855	0.031771	0.00148
21	0.020932	0.489349	0.042775	0.007989
22	0.022413	0.320036	0.070032	0.022115
23	0.021923	0.596271	0.036767	0.003018
24	1.51E-10	0.86598	1.74E-10	0
25	2.59E-10	1.59625	1.62E-10	0
26	4.49E-10	2.03979	2.20E-10	0
27	3.07E-10	1.98421	1.55E-10	0
28	1.11E-15	1.55553	7.14E-16	0
29	2.22E-15	3.34241	6.64E-16	0
30	1.11E-15	1.88913	5.88E-16	0
31	0.025167	1.98788	0.01266	3.22E-12
32	0.02556	2.05301	0.01245	1.24E-09
33	0.022521	1.74965	0.012871	1.91E-08
34	0.02203	1.81583	0.012132	1.20E-05
35	9.26E-11	1.48744	6.23E-11	0
36	NA	1.94135	NA	0
37	NA	1.22038	NA	0
38	0	1.25909	0	0
39	0	1.34506	0	0
40	1.11E-15	1.41366	7.85E-16	0
41	0	1.16717	0	0
42	0.022023	1.03237	0.021332	6.47E-05
43	0.045648	1.80734	0.025257	1.91E-05
44	0.045211	2.23276	0.020249	1.23E-05
45	0.046428	1.66757	0.027842	2.35E-05
46	0.055661	1.9622	0.028367	8.71E-08
47	0.029164	1.98204	0.014714	4.56E-08
48	8.70E-10	1.92962	4.51E-10	0
49	1.74E-10	1.95308	8.91E-11	0
50	1.42E-10	1.97476	7.21E-11	0
51	0	1.47229	0	0
52	NA	1.09864	NA	NA
53	0	1.24427	0	0
54	0	1.32196	0	0
55	NA	1.34693	NA	0
56	2.22E-15	1.46659	1.51E-15	0
57	NA	1.5124	NA	0
58	0	1.54679	0	0
59	2.22E-15	1.50101	1.48E-15	0
60	NA	1.23766	NA	NA

61	3.33E-15	1.67561	1.99E-15	NA
62	0	1.61572	0	0
63	1.11E-15	1.65705	6.70E-16	0
64	0	1.54536	0	NA
65	0	1.54981	0	NA
66	0	1.36937	0	NA
67	0	1.38707	0	NA
68	0	3.27665	0	0
69	NA	2.72684	NA	0
70	NA	1.41431	NA	0
71	0	1.55729	0	0
72	NA	1.40834	NA	0
73	NA	1.34697	NA	0
74	0	1.38474	0	0
75	NA	1.52445	NA	0
76	NA	1.65039	NA	0
77	NA	3.16336	NA	0
78	0	1.73426	0	0
79	1.11E-15	3.51953	3.15E-16	0
80	0.023071	1.73764	0.013277	2.87E-07
81	0.023045	1.64494	0.014009	5.54E-05
82	0.073514	0.913444	0.08048	0.009649
83	0.095166	0.79424	0.11982	0.025789
84	0.119929	0.858891	0.139633	0.016528
85	0.10478	0.993975	0.105415	0.030257
86	0.082306	0.4733	0.173898	0.019391
87	0.024345	1.03232	0.023583	0.00197
88	0.025487	0.67668	0.037664	0.001062
89	0	1.05985	0	0
90	NA	1.81959	NA	0
91	2.22E-15	1.83073	1.21E-15	0
92	2.22E-15	1.77249	1.25E-15	0
93	NA	1.63916	NA	0
94	NA	1.52819	NA	NA
95	NA	1.66213	NA	0
96	2.22E-15	1.81374	1.22E-15	NA
97	0	2.08767	0	0
98	1.11E-15	1.65776	6.70E-16	0
99	0.027042	2.70093	0.010012	5.27E-05
100	0.026149	0.700093	0.037351	0.002507
101	0.052208	0.800651	0.065207	0.003008
102	0.048654	1.07087	0.045434	0.000555
103	0.04778	1.44457	0.033076	0.000106
104	0.023092	1.56098	0.014793	3.63E-07
105	0.023802	1.52956	0.015561	2.02E-07
106	1.11E-15	1.07404	1.03E-15	0
107	NA	1.61846	NA	0
108	1.11E-15	2.23576	4.97E-16	0
109	1.11E-15	1.39899	7.94E-16	NA
110	0	1.53762	0	0

	111	0	0.851594	0	0
	112	0	1.60932	0	0
	113	0.022133	0.689776	0.032088	0.002188
	114	0.020952	0.816106	0.025673	0.004666
	115	0.106726	2.26533	0.047113	0.002613
	116	0.111248	1.02356	0.108687	NA
	117	0.109831	0.427619	0.256843	0.26425
	118	0.093325	0.398805	0.234011	0.125301
	119	0.068892	0.351341	0.196082	0.147147
	120	0.020096	0.590531	0.034031	0.03014
	121	0.021439	0.292618	0.073265	0.065118
	122	0.02133	0.490689	0.04347	0.011399
	123	0.021291	1.36651	0.01558	0.001615
	124	0.043256	0.869228	0.049763	0.003877
	125	0.046645	1.01383	0.046009	0.000607
	126	0.0908	1.67618	0.054171	4.45E-06
	127	0.18781	1.31781	0.142517	0.017346

Gene/Segment	Method	Ka	Ks	Ka/Ks	P-Value
Cox2					
1	GMYN	0	0.360807	0	0
2	GMYN	0	1.27294	0	0
3	GMYN	0	2.15455	0	0
4	GMYN	0.020284	2.86946	0.007069	2.36E-05
5	GMYN	0.044534	1.7564	0.025356	2.05E-05
6	GMYN	0.090582	1.66923	0.054266	3.87E-06
7	GMYN	0.128833	3.33101	0.038677	2.80E-05
8	GMYN	0.120671	1.89302	0.063745	0.000233
9	GMYN	0.092393	1.63267	0.05659	NA
10	GMYN	0.047839	1.83489	0.026072	4.44E-08
11	GMYN	0.024647	5.07665	0.004855	5.33E-07
12	GMYN	0.02573	2.01014	0.0128	1.35E-06
13	GMYN	0.030827	1.18526	0.026009	0.000367
14	GMYN	0.029202	0.647275	0.045115	0.003177
15	GMYN	0.028048	0.96501	0.029065	5.30E-05
16	GMYN	0.058326	1.49971	0.038891	9.55E-06
17	GMYN	0.024908	1.53567	0.01622	2.67E-05
18	GMYN	0.049064	1.49726	0.032769	1.60E-05
19	GMYN	0.045798	1.65574	0.02766	7.90E-09
20	GMYN	0.044738	2.3923	0.018701	2.98E-05
21	GMYN	0.020889	3.07948	0.006783	3.02E-05
22	GMYN	0.067805	6.23028	0.010883	4.45E-05
23	GMYN	0.052092	2.24834	0.023169	1.24E-05
24	GMYN	0.051322	1.72431	0.029764	0.000178
25	GMYN	0.04583	1.36312	0.033621	8.54E-05
26	GMYN	0.047851	1.85103	0.025851	9.31E-07
27	GMYN	NA	1.71498	NA	0
28	GMYN	NA	1.91698	NA	0
29	GMYN	0	1.24066	0	0

30	GMYN	1.11E-15	2.13536	5.20E-16	0
31	GMYN	NA	1.71614	NA	0
32	GMYN	0.024627	1.84757	0.01333	1.66E-07
33	GMYN	0.023003	2.08418	0.011037	4.85E-06
34	GMYN	0.022815	1.51169	0.015093	7.40E-06
35	GMYN	0.023621	1.38759	0.017023	0.000337
36	GMYN	0.023631	0.999275	0.023648	0.00043
37	GMYN	0	0.762481	0	0
38	GMYN	0	2.95109	0	0
39	GMYN	1.11E-15	2.19078	5.07E-16	0
40	GMYN	1.11E-15	1.58343	7.01E-16	0
41	GMYN	0	1.44911	0	0
42	GMYN	NA	1.30392	NA	NA
43	GMYN	0	1.36592	0	0
44	GMYN	0.02116	1.55063	0.013646	2.01E-07

Gene/Segment	Method	Ka	Ks	Ka/Ks	P-Value
Cox3					
1	GMYN	1.11E-15	1.55902	7.12E-16	0
2	GMYN	NA	1.25499	NA	0
3	GMYN	NA	0.712067	NA	0
4	GMYN	0.024636	0.932572	0.026417	0.000457
5	GMYN	0.024503	1.83769	0.013333	6.21E-05
6	GMYN	0.024616	1.01333	0.024293	3.41E-05
7	GMYN	0.025822	2.20351	0.011719	3.45E-05
8	GMYN	0.025731	1.08937	0.02362	3.70E-05
9	GMYN	0.044884	1.3814	0.032492	0.002556
10	GMYN	0.044999	1.43547	0.031348	0.002117
11	GMYN	0.048917	1.13975	0.042919	0.000237
12	GMYN	0.047127	1.16133	0.04058	0.000488
13	GMYN	0.042663	1.63143	0.02615	0.000166
14	GMYN	2.59E-10	1.66869	1.55E-10	0
15	GMYN	3.16E-10	1.61669	1.95E-10	0
16	GMYN	2.91E-10	1.65865	1.75E-10	0
17	GMYN	4.17E-10	1.57722	2.65E-10	NA
18	GMYN	2.27E-10	1.60962	1.41E-10	NA
19	GMYN	0	1.55059	0	0
20	GMYN	0	2.63022	0	0
21	GMYN	2.05E-10	1.5316	1.34E-10	0
22	GMYN	4.07E-10	1.60475	2.53E-10	0
23	GMYN	3.69E-10	1.52492	2.42E-10	NA
24	GMYN	1.98E-10	1.22053	1.62E-10	NA
25	GMYN	1.40E-10	1.30128	1.07E-10	NA
26	GMYN	0.045307	1.58156	0.028647	2.30E-06
27	GMYN	0.045854	1.87369	0.024472	8.76E-06
28	GMYN	0.053139	3.14021	0.016922	2.34E-07
29	GMYN	0.08405	2.05694	0.040862	4.94E-07
30	GMYN	0.08048	1.82509	0.044097	5.76E-09
31	GMYN	0.024395	1.81388	0.013449	8.19E-08

32	GMYN	0.0202	1.41143	0.014312	NA
33	GMYN	0.020053	1.37495	0.014585	3.43E-07
34	GMYN	0.02131	1.66279	0.012816	1.32E-06
35	GMYN	0.021372	1.67666	0.012747	8.79E-08
36	GMYN	0.02105	1.6154	0.013031	6.20E-07
37	GMYN	0.02306	1.89197	0.012188	3.23E-08
38	GMYN	0.023669	1.95726	0.012093	1.23E-08
39	GMYN	0.022161	1.77726	0.012469	NA
40	GMYN	0.023481	1.90886	0.012301	3.15E-09
41	GMYN	0.026219	2.19491	0.011945	4.45E-07
42	GMYN	0.025495	3.057	0.00834	1.82E-06
43	GMYN	0.023933	1.59473	0.015008	9.99E-07
44	GMYN	0.024398	1.66315	0.01467	5.39E-09
45	GMYN	0.022928	1.69358	0.013538	7.57E-09
46	GMYN	0.022081	1.78702	0.012357	1.95E-09
47	GMYN	0	1.63953	0	NA
48	GMYN	NA	1.71447	NA	0
49	GMYN	NA	0.895338	NA	0
50	GMYN	1.11E-15	1.82835	6.07E-16	0

Gene/Segment	Method	Ka	Ks	Ka/Ks	P-Value
CyB					
1	GMYN	NA	0.699305	NA	0
2	GMYN	2.22E-15	0.824129	2.69E-15	0
3	GMYN	0.019148	1.03016	0.018588	0.000508
4	GMYN	0.018705	0.556265	0.033626	0.092974
5	GMYN	0.061801	0.92864	0.06655	0.051627
6	GMYN	0.092787	0.667418	0.139024	0.02753
7	GMYN	0.075435	1.02355	0.0737	0.000752
8	GMYN	0.075382	1.09099	0.069095	0.00065
9	GMYN	0.077445	1.22838	0.063046	0.000378
10	GMYN	0.021688	1.72015	0.012608	2.07E-07
11	GMYN	6.95E-11	1.55343	4.47E-11	0
12	GMYN	5.91E-11	1.9157	3.08E-11	0
13	GMYN	0.019602	0.719334	0.02725	0.013343
14	GMYN	0.020312	0.308495	0.065842	0.138275
15	GMYN	0.021252	0.184461	0.115209	0.204629
16	GMYN	0.043855	0.942085	0.046551	0.022813
17	GMYN	0.049261	0.465083	0.105918	0.029267
18	GMYN	0.045857	0.93142	0.049233	0.003895
19	GMYN	0.046565	2.06102	0.022593	0.000103
20	GMYN	0.043499	1.56009	0.027883	NA
21	GMYN	0.022668	1.71893	0.013187	NA
22	GMYN	0.02236	1.67554	0.013345	NA
23	GMYN	0.023488	1.75524	0.013381	3.69E-08
24	GMYN	0.023158	1.5965	0.014505	2.32E-08
25	GMYN	0.050061	1.17445	0.042625	0.000493
26	GMYN	0.054617	0.692192	0.078904	0.002045
27	GMYN	0.04909	0.828081	0.059281	0.000943

28	GMYN	0.026168	0.82673	0.031652	0.000453
29	GMYN	0.023952	1.08454	0.022085	0.00323
30	GMYN	NA	0.733319	NA	0
31	GMYN	1.11E-15	0.368792	3.01E-15	0
32	GMYN	0.021968	0.143104	0.153511	0.248479
33	GMYN	0.049486	0.523647	0.094503	0.022825
34	GMYN	0.05229	1.05447	0.049589	0.001247
35	GMYN	0.078814	1.17447	0.067106	0.000858
36	GMYN	0.076038	1.18653	0.064085	0.000114
37	GMYN	0.072838	1.48965	0.048896	5.08E-05
38	GMYN	0.045886	1.37842	0.033289	0.000499
39	GMYN	0.044009	0.473941	0.092858	0.032063
40	GMYN	0.022471	0.869692	0.025838	0.000126
41	GMYN	0.024789	0.844984	0.029337	5.20E-05
42	GMYN	1.49E-10	2.77458	5.36E-11	0
43	GMYN	0.023937	1.99588	0.011993	2.70E-09
44	GMYN	0.025971	1.89474	0.013707	1.54E-07
45	GMYN	0.023011	1.85588	0.012399	4.01E-06
46	GMYN	0.021797	1.87198	0.011644	4.07E-06
47	GMYN	4.52E-11	1.49277	3.02E-11	0
48	GMYN	0.023454	1.75753	0.013345	6.53E-07
49	GMYN	0.023219	1.72502	0.01346	1.75E-08
50	GMYN	0.021936	1.50973	0.014529	NA
51	GMYN	0.021523	1.42269	0.015128	NA
52	GMYN	NA	1.69621	NA	0
53	GMYN	0	2.02627	0	0
54	GMYN	0	1.7408	0	0
55	GMYN	1.11E-15	0.995416	1.12E-15	0
56	GMYN	0.023372	1.28946	0.018126	0.000521
57	GMYN	0.022865	0.898194	0.025457	0.002164
58	GMYN	0.020694	1.18159	0.017514	NA
59	GMYN	0.021704	1.46876	0.014777	2.13E-06
60	GMYN	0.024644	1.90123	0.012962	7.25E-08
61	GMYN	3.33E-15	1.90031	1.75E-15	0
62	GMYN	NA	1.5617	NA	0
63	GMYN	NA	1.85446	NA	0
64	GMYN	NA	2.27223	NA	0
65	GMYN	NA	0.733911	NA	0
66	GMYN	1.11E-15	0.677045	1.64E-15	0
67	GMYN	NA	0.824196	NA	0
68	GMYN	2.22E-15	0.573541	3.87E-15	0
69	GMYN	0	0.679452	0	0
70	GMYN	0.026135	0.907673	0.028794	0.000541
71	GMYN	0.051603	1.92741	0.026773	6.08E-06
72	GMYN	0.047555	1.63426	0.029099	5.48E-06
73	GMYN	0.045391	1.29688	0.035	NA
74	GMYN	0.044349	1.32623	0.03344	NA
75	GMYN	0.020746	1.20125	0.01727	7.37E-05
76	GMYN	1.11E-15	1.55145	7.16E-16	0
77	GMYN	1.11E-15	1.39236	7.97E-16	0

78	GMYN	NA	1.61843	NA	0
79	GMYN	1.11E-15	2.06769	5.37E-16	0
80	GMYN	0.024442	2.40898	0.010146	1.13E-07
81	GMYN	0.021353	1.67835	0.012723	2.89E-09
82	GMYN	0.020993	1.61068	0.013034	5.74E-07
83	GMYN	0.022986	0.998646	0.023017	3.63E-05
84	GMYN	0.049814	2.514	0.019815	0.000696
85	GMYN	0.04675	0.772365	0.060529	0.00075
86	GMYN	0.055561	0.796073	0.069794	0.00282
87	GMYN	0.129529	1.04607	0.123824	0.00047

Gene/Segment	Method	Ka	Ks	Ka/Ks	P-Value
NAD1					
1	GMYN	0.154148	1.12747	0.136721	0.036493
2	GMYN	0.091034	1.66389	0.054712	0.000626
3	GMYN	0.063481	1.47442	0.043055	1.90E-05
4	GMYN	0.042366	1.50964	0.028064	1.05E-05
5	GMYN	0.073396	1.15968	0.06329	0.000447
6	GMYN	0.070298	1.54332	0.04555	4.76E-05
7	GMYN	0.045684	1.12985	0.040433	0.000674
8	GMYN	0.04557	1.19145	0.038248	0.000147
9	GMYN	0.04782	0.87081	0.054915	0.000461
10	GMYN	3.33E-15	0.955061	3.49E-15	0
11	GMYN	NA	1.08428	NA	0
12	GMYN	0.023851	0.962762	0.024774	4.28E-05
13	GMYN	0.04865	1.83351	0.026534	1.69E-05
14	GMYN	0.050161	1.47176	0.034082	2.79E-05
15	GMYN	0.047045	3.8708	0.012154	6.77E-06
16	GMYN	0.093887	3.72881	0.025179	0.000539
17	GMYN	0.103182	1.70723	0.060438	8.07E-06
18	GMYN	0.091619	1.57108	0.058316	NA
19	GMYN	0.090443	1.35147	0.066922	NA
20	GMYN	0.101448	1.85281	0.054754	9.95E-07
21	GMYN	0.071472	1.66428	0.042945	NA
22	GMYN	0.09188	1.70723	0.053818	4.43E-07
23	GMYN	0.098098	1.92393	0.050988	5.21E-06
24	GMYN	0.125132	1.90625	0.065643	9.19E-07
25	GMYN	0.149215	1.73257	0.086124	5.65E-06
26	GMYN	0.07286	3.15731	0.023077	5.59E-06
27	GMYN	0.070023	1.74462	0.040137	5.52E-06
28	GMYN	0.021114	1.54493	0.013667	4.30E-06
29	GMYN	0.047602	2.0614	0.023092	3.11E-05
30	GMYN	0.079976	2.15106	0.03718	9.24E-05
31	GMYN	0.098481	1.89909	0.051857	0.001229
32	GMYN	0.096653	1.3636	0.070881	0.005563
33	GMYN	0.09357	1.1615	0.08056	0.015616
34	GMYN	0.062635	0.409542	0.152939	0.103061
35	GMYN	NA	0.723783	NA	0
36	GMYN	0.063796	1.13362	0.056276	0.003841

37	GMYN	0.119137	1.38156	0.086234	0.021658
38	GMYN	0.187739	0.872826	0.215094	0.03992
39	GMYN	0.21581	1.69129	0.1276	1.57E-05
40	GMYN	0.234959	1.76557	0.133078	9.05E-05
41	GMYN	0.301168	1.79069	0.168185	0.004207
42	GMYN	0.276921	1.75106	0.158145	9.60E-06
43	GMYN	0.263628	1.58143	0.166702	0.000686
44	GMYN	0.172271	1.44615	0.119124	0.000399
45	GMYN	0.139429	1.96128	0.071091	0.000676
46	GMYN	0.044828	1.63556	0.027408	NA
47	GMYN	0.021312	1.64781	0.012933	NA
48	GMYN	0.021396	3.59604	0.00595	1.75E-06
49	GMYN	0.022629	1.86165	0.012155	7.13E-10
50	GMYN	0.023358	1.94127	0.012032	4.43E-10
51	GMYN	NA	2.00881	NA	0
52	GMYN	7.77E-11	1.82757	4.25E-11	0
53	GMYN	5.21E-11	1.61937	3.22E-11	NA
54	GMYN	3.66E-11	1.39093	2.63E-11	NA
55	GMYN	2.95E-11	1.49302	1.97E-11	0
56	GMYN	1.68E-11	1.35581	1.24E-11	NA
57	GMYN	0	1.37037	0	0
58	GMYN	0.134272	1.70524	0.078741	1.95E-05
59	GMYN	0.228007	2.44482	0.093261	0.002794
60	GMYN	0.258546	1.35092	0.191385	NA
61	MYN	0.408767	0.992635	0.411799	0.131166
62	GMYN	0.324351	1.8136	0.178844	0.076587
63	GMYN	0.172185	3.32968	0.051712	0.000189
64	GMYN	0.080435	1.43642	0.055997	1.53E-05
65	GMYN	0.049744	0.921516	0.05398	NA
66	GMYN	0.061111	1.22089	0.050054	1.63E-05
67	GMYN	0.040117	0.974469	0.041168	NA
68	GMYN	0.129475	2.68281	0.048261	0.003948
69	GMYN	0.193176	0.922314	0.209447	0.029006
70	GMYN	0.166975	0.332987	0.501445	0.274717

Gene/Segment	Method	Ka	Ks	Ka/Ks	P-Value
NAD2					
1	GMYN	0.183765	0.485844	0.378238	0.232426
2	GMYN	0.215979	1.45701	0.148234	NA
3	GMYN	0.163801	4.1685	0.039295	0.000159
4	GMYN	0.179352	1.7503	0.102469	0.000752
5	GMYN	0.19207	2.4062	0.079823	0.000531
6	GMYN	0.220502	1.79365	0.122935	0.029377
7	GMYN	0.136138	0.451945	0.301227	0.142047
8	GMYN	0.189192	1.18892	0.15913	0.005265
9	GMYN	0.198838	2.24984	0.088379	0.019166
10	GMYN	0.227101	0.544506	0.417078	0.372548
11	GMYN	0.128406	1.26871	0.10121	0.000698

12	GMYN	0.130904	1.59557	0.082042	0.000156
13	GMYN	0.105041	1.0226	0.10272	0.001449
14	GMYN	0.050136	1.46038	0.034331	2.77E-05
15	GMYN	0.070353	1.82033	0.038648	0.000186
16	GMYN	0.120832	1.61349	0.074889	0.013504
17	GMYN	0.122849	2.27213	0.054068	0.011482
18	GMYN	0.138348	0.728749	0.189843	0.054878
19	GMYN	0.14558	1.10474	0.131778	0.013218
20	GMYN	0.149404	0.903505	0.165361	0.012642
21	GMYN	0.093617	1.19662	0.078234	0.003114
22	GMYN	0.099521	1.81577	0.054809	0.000405
23	GMYN	0.096411	1.5278	0.063105	7.70E-06
24	GMYN	0.119615	1.05029	0.113887	NA
25	GMYN	0.143426	0.997001	0.143858	NA
26	GMYN	0.182078	1.41527	0.128652	0.003592
27	GMYN	0.127458	2.28713	0.055728	0.018398
28	GMYN	0.103121	0.801545	0.128653	0.017503
29	GMYN	0.048499	0.876153	0.055355	0.001122
30	GMYN	0.070163	0.509442	0.137726	0.026912
31	GMYN	0.16608	0.487874	0.340416	0.193895
32	GMYN	0.247058	1.61507	0.152971	0.025587
33	GMYN	0.351188	1.03474	0.339396	0
34	GMYN	0.47491	1.47059	0.322938	0.011995
35	GMYN	0.408046	1.43784	0.283791	NA
36	GMYN	0.245533	1.95461	0.125617	4.49E-06
37	GMYN	0.26963	1.87351	0.143917	NA
38	GMYN	0.184384	1.95213	0.094453	9.52E-08
39	GMYN	0.151758	1.9527	0.077717	6.07E-06
40	GMYN	0.123317	2.10516	0.058578	3.82E-05
41	GMYN	0.259266	4.72355	0.054888	8.28E-05
42	GMYN	0.146668	1.64601	0.089105	0.00019
43	GMYN	0.232708	1.75223	0.132807	0.033131
44	GMYN	0.210073	1.95369	0.107527	0.001374
45	GMYN	0.203845	1.28645	0.158455	0.052902
46	GMYN	0.172824	0.899807	0.192068	0.132675
47	GMYN	0.142408	0.30446	0.467739	0.456307
48	GMYN	0.146385	0.963699	0.151899	0.009451
49	GMYN	0.15205	2.83871	0.053563	0.004838
50	GMYN	0.155664	1.57347	0.09893	0.007553
51	GMYN	0.262128	1.24611	0.210358	NA
52	GMYN	0.330529	0.507117	0.651779	0.465373
53	GMYN	0.265727	0.653273	0.406763	0.328947
54	GMYN	0.234368	2.31925	0.101053	0.016662
55	GMYN	0.173298	0.859787	0.201559	0.085188
56	GMYN	0.069878	1.04467	0.06689	0.003536
57	GMYN	0.09676	1.10313	0.087714	0.005874
58	GMYN	0.15184	1.07196	0.141646	0.015028
59	GMYN	0.18386	0.923181	0.199159	0.0153
60	GMYN	0.215956	1.69546	0.127373	0.005467
61	GMYN	0.199891	1.52011	0.131498	NA

62	GMYN	0.133655	1.34693	0.099229	NA
63	GMYN	0.108193	1.38092	0.078349	NA
64	GMYN	0.132637	1.64019	0.080867	0.000178
65	GMYN	0.116142	2.852	0.040723	0.000365
66	GMYN	0.069317	1.05173	0.065907	0.000623
67	GMYN	0.15131	0.504387	0.299988	0.190062
68	GMYN	0.16541	0.337957	0.489441	0.426159

Gene/Segment	Method	Ka	Ks	Ka/Ks	P-Value
NAD3					
1	GMYN	0.13697	0.785846	0.174296	0.019078
2	GMYN	0.166072	0.906145	0.183273	0.020913
3	GMYN	0.165454	0.840488	0.196855	0.02585
4	GMYN	0.078637	1.17127	0.067139	0.000846
5	GMYN	0.048477	1.01342	0.047835	0.000359
6	GMYN	NA	2.08627	NA	0
7	GMYN	0	1.93302	0	0
8	GMYN	NA	1.37955	NA	0
9	GMYN	2.22E-15	1.86586	1.19E-15	0
10	GMYN	NA	1.11982	NA	0
11	GMYN	0	1.1792	0	0
12	GMYN	4.60E-10	3.44446	1.33E-10	0
13	GMYN	2.27E-10	1.8566	1.22E-10	0
14	GMYN	1.11E-09	2.37981	4.65E-10	0
15	GMYN	5.78E-10	1.71083	3.38E-10	NA
16	GMYN	5.43E-10	1.85886	2.92E-10	NA
17	GMYN	2.65E-10	1.96045	1.35E-10	0
18	GMYN	2.73E-10	2.04833	1.33E-10	0
19	GMYN	0.025751	2.06628	0.012463	2.12E-08
20	GMYN	0.077637	1.35741	0.057195	6.10E-05
21	GMYN	0.102878	1.59488	0.064505	0.000114
22	GMYN	0.192484	0.452952	0.424954	0.494493
23	GMYN	0.260287	1.73218	0.150266	0.000111
24	GMYN	0.199456	0.892577	0.22346	0.052169
25	GMYN	0.097434	1.70729	0.057069	1.27E-05

Gene/Segment	Method	Ka	Ks	Ka/Ks	P-Value
NAD5					
1	GMYN	0.144358	0.510971	0.282518	0.050466
2	GMYN	0.13264	1.10906	0.119598	NA
3	GMYN	0.130916	1.03582	0.126388	NA
4	GMYN	0.110115	1.10402	0.09974	NA
5	GMYN	0.154904	1.34247	0.115387	NA
6	GMYN	0.12648	1.47716	0.085624	4.46E-06
7	GMYN	0.15237	1.65733	0.091937	1.76E-05
8	GMYN	0.222966	1.52947	0.14578	9.78E-05
9	GMYN	0.208237	4.5563	0.045703	0.000429

10	GMYN	0.135866	1.20599	0.112659	0.022815
11	GMYN	0.060138	1.3646	0.04407	0.005277
12	GMYN	0.062577	1.43019	0.043754	9.92E-06
13	GMYN	0.04013	1.25143	0.032068	NA
14	GMYN	0.044059	1.64201	0.026833	4.16E-06
15	GMYN	0.022441	1.83293	0.012243	1.23E-07
16	GMYN	0.02177	2.04394	0.010651	3.37E-06
17	GMYN	0.021139	1.75739	0.012029	1.31E-05
18	GMYN	1.11E-15	1.36186	8.15E-16	0
19	GMYN	NA	1.28381	NA	0
20	GMYN	NA	1.36956	NA	0
21	GMYN	NA	0.402032	NA	0
22	GMYN	0.021942	1.05062	0.020884	6.06E-05
23	GMYN	0.022007	1.46459	0.015026	6.57E-05
24	GMYN	0.021731	1.13846	0.019088	0.000361
25	GMYN	0.020377	2.11204	0.009648	3.19E-05
26	GMYN	0	1.50044	0	0
27	GMYN	1.11E-15	1.7097	6.49E-16	0
28	GMYN	4.58E-11	1.08967	4.20E-11	NA
29	GMYN	4.45E-11	1.34783	3.30E-11	0
30	GMYN	2.84E-11	1.51051	1.88E-11	NA
31	GMYN	0.021959	1.42851	0.015372	NA
32	GMYN	0.023315	1.62958	0.014307	5.07E-08
33	GMYN	0.02191	1.23434	0.01775	3.42E-05
34	GMYN	0.093129	1.23989	0.075111	0.000807
35	GMYN	0.213523	0.468024	0.456221	0.192429
36	GMYN	0.281013	0.704331	0.398979	0.150364
37	GMYN	0.295803	2.59486	0.113996	0.023874
38	GMYN	0.208559	1.62508	0.128338	2.95E-05
39	GMYN	0.171432	1.77703	0.096471	2.56E-05
40	GMYN	0.125978	3.02389	0.041661	1.16E-05
41	GMYN	0.070532	1.63436	0.043156	6.30E-07
42	GMYN	0.021525	1.46316	0.014711	1.40E-06
43	GMYN	0.022544	2.12803	0.010594	3.06E-06
44	GMYN	0.023324	1.58392	0.014726	5.44E-07
45	GMYN	NA	1.29526	NA	0
46	GMYN	0.023941	1.37312	0.017436	5.16E-05
47	GMYN	0.022964	1.59786	0.014372	0.000148
48	GMYN	0.154256	1.60294	0.096233	0.00829
49	GMYN	0.258783	1.34857	0.191894	0.024531
50	GMYN	0.455773	1.03618	0.439859	0.245042
51	MYN	0.462726	1.52177	0.304071	0.253585
52	MYN	0.422351	2.22039	0.190215	0.007288
53	MYN	0.376481	2.41678	0.155778	0.022762
54	GMYN	0.256439	1.50484	0.17041	0.000184
55	GMYN	0.160778	1.40309	0.114588	NA
56	GMYN	0.104223	2.37424	0.043897	6.95E-05
57	GMYN	0.04263	1.75605	0.024276	0.000336
58	GMYN	0.021152	1.62744	0.012997	7.55E-07
59	GMYN	3.35E-11	1.75392	1.91E-11	0

60	GMYN	3.34E-11	1.66246	2.01E-11	0
61	GMYN	0.067041	1.64684	0.040709	NA
62	GMYN	0.068382	1.74618	0.039161	1.33E-08
63	GMYN	0.095837	1.84423	0.051966	4.25E-08
64	GMYN	0.096067	1.84838	0.051974	NA
65	GMYN	0.114347	1.66912	0.068507	NA
66	GMYN	0.043978	1.70448	0.025802	3.27E-08
67	GMYN	0.022758	1.19721	0.019009	9.79E-05
68	GMYN	0.021334	0.77448	0.027546	0.001121
69	GMYN	0.022707	0.470118	0.048301	0.005477
70	GMYN	0.046632	0.79935	0.058338	0.000751
71	GMYN	0.047656	1.01845	0.046793	0.000205
72	GMYN	0.127959	1.82618	0.070069	1.76E-05
73	GMYN	0.207519	2.04475	0.101489	0.001387
74	GMYN	0.217177	1.52132	0.142756	0.000125
75	GMYN	0.321455	1.61504	0.199039	0
76	GMYN	0.315541	1.32937	0.237361	NA
77	GMYN	0.186803	1.48998	0.125373	NA
78	GMYN	0.104299	1.57046	0.066413	NA
79	GMYN	0.082653	1.73442	0.047655	2.78E-07
80	GMYN	0.022296	2.17969	0.010229	1.17E-06
81	GMYN	0.097279	1.21895	0.079805	0.002632
82	GMYN	0.068281	3.50002	0.019509	2.45E-05
83	GMYN	0.095309	1.06855	0.089194	0.002252
84	GMYN	0.227026	2.60538	0.087137	0.004205
85	GMYN	0.187828	3.50907	0.053526	0.001875
86	GMYN	0.14111	1.65	0.085522	0.000193
87	GMYN	0.142443	2.85732	0.049852	0.000348
88	GMYN	0.165834	2.54893	0.06506	0.000787
89	GMYN	0.20233	0.992545	0.203849	0.138192
90	GMYN	0.219861	0.666256	0.329995	0.118348
91	GMYN	0.231372	0.573895	0.403161	0.113271
92	GMYN	0.198571	1.47882	0.134276	0.000648
93	GMYN	0.135757	1.32095	0.102773	0.000457
94	GMYN	0.104837	0.884534	0.118522	NA
95	GMYN	0.138033	1.21643	0.113474	NA
96	GMYN	0.171486	1.51694	0.113048	0
97	GMYN	0.260191	1.4774	0.176115	NA
98	GMYN	0.260269	2.09764	0.124077	0.024258
99	GMYN	0.283126	0.623468	0.454114	0.544547
100	GMYN	0.277408	0.675554	0.410638	0.083031
101	GMYN	0.299816	1.87364	0.160018	0.000209
102	GMYN	0.241549	1.90895	0.126535	1.26E-05
103	GMYN	0.298349	1.51319	0.197166	0.004855
104	GMYN	0.311879	1.48523	0.209988	0.020342
105	GMYN	0.319608	0.81788	0.390776	0.107998
106	GMYN	0.197344	0.582167	0.338982	0.037403
107	GMYN	0.181869	0.518867	0.350512	0.101658
108	GMYN	0.1074	0.677681	0.158482	0.029716
109	GMYN	0.074075	1.85338	0.039968	0.000946

110	GMYN	0.092486	1.63909	0.056425	3.40E-05
111	GMYN	0.193315	1.58075	0.122294	1.34E-05
112	GMYN	0.272257	1.43544	0.189668	NA
113	GMYN	0.466367	0.98419	0.473859	0.28968
114	GMYN	0.332136	0.535967	0.619694	0.374581
115	GMYN	0.265748	1.51373	0.175558	0.00029
116	GMYN	0.112209	1.19123	0.094196	0.003749
117	GMYN	0.099898	0.732739	0.136335	0.052995
118	GMYN	0.143351	2.95879	0.048449	0.001259
119	GMYN	0.257961	1.62204	0.159035	0.010701
120	GMYN	0.330454	0.738955	0.447191	0.156977
121	GMYN	0.323563	1.37426	0.235445	NA
122	GMYN	0.245929	1.47867	0.166318	9.74E-05
123	GMYN	0.163435	1.4324	0.114099	NA
124	GMYN	0.212973	1.85777	0.114639	1.93E-05

Gene/Segment	Method	Ka	Ks	Ka/Ks	P-Value
NAD6					
1	GMYN	0.085457	1.53704	0.055599	2.27E-06
2	GMYN	0.082801	1.55594	0.053216	3.20E-06
3	GMYN	0.081275	1.64467	0.049417	0
4	GMYN	0.068797	1.69476	0.040594	2.19E-06
5	GMYN	0.072023	1.87161	0.038482	4.70E-07
6	GMYN	0.077411	1.98152	0.039067	6.29E-07
7	GMYN	0.140326	1.97385	0.071092	5.47E-06
8	GMYN	0.129648	1.57432	0.082352	0.000201
9	GMYN	0.101728	1.98806	0.05117	2.59E-06
10	GMYN	0.093391	1.92792	0.048441	1.53E-07
11	GMYN	0.025533	1.95395	0.013067	1.13E-08
12	GMYN	0.02681	2.05211	0.013064	1.20E-09
13	GMYN	0.025427	2.04047	0.012461	1.03E-08
14	GMYN	1.11E-15	1.16479	9.53E-16	0
15	GMYN	NA	1.67802	NA	0
16	GMYN	0.022578	1.85629	0.012163	1.98E-07
17	GMYN	0.091032	1.30323	0.069851	0.000725
18	GMYN	0.238569	1.13674	0.209871	0.015565
19	GMYN	0.268836	0.519635	0.517356	0.370159
20	GMYN	0.258227	0.534658	0.482976	0.146336
21	GMYN	0.324345	0.591361	0.548472	0.368703
22	GMYN	0.253728	1.23685	0.205141	0.001261
23	GMYN	0.231407	1.06152	0.217997	NA
24	GMYN	0.220647	1.53544	0.143703	0.000243
25	GMYN	0.204961	2.72753	0.075145	0.002982
26	GMYN	0.207224	1.22116	0.169695	NA
27	GMYN	0.120202	1.75481	0.068499	4.69E-05
28	GMYN	0.148056	1.40872	0.1051	0.001168
29	GMYN	0.202654	2.13502	0.094919	0.001243

30	GMYN	0.212715	1.45726	0.145969	0.002336
31	GMYN	0.156912	1.50484	0.104272	0.000504
32	GMYN	0.095413	1.89465	0.050359	0.00098
33	GMYN	0.023162	2.07495	0.011163	0.000175

Gene/Segment	Method	Ka	Ks	Ka/Ks	P-Value
ND4					
1	GMYN	0.088084	0.870248	0.101216	0.001581
2	GMYN	0.058824	0.758454	0.077558	0.001226
3	GMYN	0.135203	1.85997	0.072691	0.001525
4	GMYN	0.250008	0.734145	0.340543	0.048447
5	GMYN	0.212025	1.45799	0.145423	0.00812
6	GMYN	0.218917	2.3648	0.092573	0.002533
7	GMYN	0.195538	1.78143	0.109765	0.000453
8	GMYN	0.189573	2.47895	0.076473	0.00134
9	GMYN	0.169338	1.33075	0.127251	NA
10	GMYN	0.129825	1.52165	0.085319	0
11	GMYN	0.209065	4.5848	0.0456	0.017265
12	GMYN	0.163785	1.4031	0.116731	0.000851
13	GMYN	0.08955	2.11918	0.042257	0.000243
14	GMYN	0.065882	1.45492	0.045282	1.43E-05
15	GMYN	0.069458	1.33316	0.052101	NA
16	GMYN	0.021458	1.1746	0.018268	NA
17	GMYN	3.44E-10	1.52968	2.25E-10	NA
18	GMYN	5.01E-10	1.75203	2.86E-10	NA
19	GMYN	0.026652	2.84687	0.009362	2.51E-07
20	GMYN	0.081038	1.84213	0.043992	1.07E-08
21	GMYN	0.228617	2.06325	0.110805	0.004777
22	GMYN	0.320993	1.32254	0.24271	0.00824
23	GMYN	0.586226	1.63405	0.358757	0.065924
24	GMYN	0.561508	1.95875	0.286666	0.066495
25	GMYN	0.523629	1.31173	0.399189	0.166454
26	GMYN	0.369081	1.47252	0.250645	0.13756
27	GMYN	0.215098	0.580441	0.370577	0.128144
28	GMYN	0.046841	0.526952	0.08889	0.018855
29	GMYN	0	1.09831	0	0
30	GMYN	1.11E-15	1.36095	8.16E-16	0
31	GMYN	0.021933	1.50769	0.014548	3.67E-06
32	GMYN	0.062962	1.36971	0.045968	5.53E-06
33	GMYN	0.092798	1.65945	0.055921	4.67E-06
34	GMYN	0.085749	1.32723	0.064607	0
35	GMYN	0.100366	1.59416	0.062958	2.20E-05
36	GMYN	0.102663	1.66745	0.061569	3.28E-06
37	GMYN	0.062613	1.43925	0.043504	NA
38	GMYN	0.045176	1.5652	0.028863	1.73E-06
39	GMYN	0.145369	1.56989	0.092599	8.19E-05
40	GMYN	0.203933	1.14791	0.177656	0.006329
41	GMYN	0.221771	0.637724	0.347754	0.036372

42	GMYN	0.188893	0.680501	0.277579	0.025232
43	GMYN	0.19568	0.682484	0.286717	0.040021
44	GMYN	0.087541	0.850291	0.102954	0.005154
45	GMYN	0.04356	0.437113	0.099655	0.024549
46	GMYN	0.022824	0.755394	0.030215	0.012941
47	GMYN	0.04995	1.07413	0.046503	0.000161
48	GMYN	0.025341	1.12463	0.022533	1.83E-05
49	GMYN	0.022903	1.69177	0.013538	1.89E-07
50	GMYN	0.069583	1.8276	0.038074	1.54E-07
51	GMYN	0.160422	1.72993	0.092734	2.07E-07
52	GMYN	0.190506	2.65945	0.071634	0.000114
53	GMYN	0.206928	2.48447	0.083289	0.000592
54	GMYN	0.184639	1.28014	0.144234	0.001604
55	GMYN	0.105154	1.30749	0.080425	0.000218
56	GMYN	0.062354	1.63939	0.038035	0.001697
57	GMYN	0.019966	2.69659	0.007404	0.000191
58	GMYN	0.043082	2.50273	0.017214	0.001595
59	GMYN	0.021916	2.01563	0.010873	0.000848
60	GMYN	0.022423	1.30232	0.017218	0.002534
61	GMYN	0.069547	0.689444	0.100874	0.032023
62	GMYN	0.121701	0.804515	0.151272	0.061337
63	GMYN	0.121657	0.813116	0.149618	0.014359
64	GMYN	0.191445	1.30941	0.146207	0.001514
65	GMYN	0.212303	1.43716	0.147724	0.028012
66	GMYN	0.126626	2.15221	0.058835	0.002035
67	GMYN	0.073054	0.953167	0.076643	0.001716
68	GMYN	0.100897	1.30839	0.077115	0.011128
69	GMYN	0.04819	0.427312	0.112775	0.025868
70	GMYN	0.068839	0.36457	0.188823	0.067223
71	GMYN	0.121134	0.344457	0.351666	0.16864
72	GMYN	0.157829	0.867841	0.181864	0.021237
73	GMYN	0.178103	1.32544	0.134373	0.002746
74	GMYN	0.232055	0.994979	0.233226	0.024395
75	GMYN	0.215257	1.94701	0.110558	0.000838
76	GMYN	0.264311	2.00101	0.132089	0.000292
77	GMYN	0.199088	1.99553	0.099767	5.77E-05
78	GMYN	0.14526	1.0999	0.132067	0.000642
79	GMYN	0.181328	1.35086	0.134231	0.002202
80	GMYN	0.252014	0.808723	0.31162	0.088034
81	GMYN	0.22777	0.544752	0.418116	0.230774
82	GMYN	0.273546	0.674421	0.405602	0.236025
83	GMYN	0.299012	1.71585	0.174265	0.001096
84	GMYN	0.169974	1.42957	0.118899	0
85	GMYN	0.13689	1.48503	0.09218	NA
86	GMYN	0.102531	0.997206	0.102818	NA

Gene/Segment	Method	Ka	Ks	Ka/Ks	P-Value
ND4L					
1	GMYN	0.150196	1.24798	0.120351	0
2	GMYN	0.087919	1.20186	0.073153	0.009638
3	GMYN	0.021082	1.09348	0.019279	0.000132
4	GMYN	0.099607	2.40374	0.041438	0.00247
5	GMYN	0.194993	1.69321	0.115162	0.000426
6	GMYN	0.151022	1.72658	0.087469	0.00235
7	GMYN	0.25161	1.52425	0.165071	0.002858
8	GMYN	0.261035	1.16934	0.223234	0.042202
9	GMYN	0.159978	1.07501	0.148815	0.004822
10	GMYN	0.09117	0.487696	0.186941	0.007272
11	GMYN	0.140026	2.03506	0.068807	0.000136
12	GMYN	0.068553	1.66892	0.041076	1.31E-06
13	GMYN	0.069858	1.73806	0.040193	4.91E-06
14	GMYN	0.129499	1.76669	0.0733	4.83E-07
15	GMYN	0.12804	1.77304	0.072215	9.77E-06
