# Supplementary material for: The complete mitochondrial genome and description of a new cryptic species of Benedenia Diesing, 1858 (Monogenea: Capsalidae), a major pathogen infecting the yellowtail kingfish Seriola lalandi Valenciennes in the South-East Pacific
Source: Parasit Vectors. 2019 Oct 17;12:490. doi: 10.1186/s13071-019-3711-5 (PMC6798380; doi:10.1186/s13071-019-3711-5)
Supplement: Supplementary file 2 — Additional file 2: Figure S1. Secondary structure prediction analysis of the non-coding putative D-loop/CR in the mitochondrial genome of B. humboldti n. sp. from the SEP. [file 13071_2019_3711_MOESM2_ESM.pdf]

The complete mitochondrial genome and description of a new cryptic species of *Benedenia* Diesing, 1858 (Monogenea: Capsalidae), a major pathogen infecting the yellowtail kingfish *Seriola lalandi* Valenciennes, 1833 in the South-East Pacific

J. Antonio Baeza[1, 2, 3,*]

Fabiola Sepulveda[4]

Teresa Gonzalez[4,*]

[1] *Department of Biological Sciences, 132 Long Hall, Clemson University, Clemson, SC 29634, USA.*

[2] *Smithsonian Marine Station at Fort Pierce, 701 Seaway Drive, Fort Pierce, Florida 34949, USA.*

[3] *Departamento de Biología Marina, Facultad de Ciencias del Mar, Universidad Católica del Norte, Larrondo 1281, Coquimbo, Chile.*

[4] *Laboratorio Eco-parasitologia y Epidemiologia Marina (LEPyEM), Instituto de Ciencias Naturales Alexander von Humboldt, Facultad de Ciencias del Mar y Recursos Biologicos, Universidad de Antofagasta, Angamos 601, Antofagasta, Chile.*

Additional file 2: Figure S1. Secondary structure prediction analysis of the non coding putative D-loop/CR in the mitochondrial genome of *Benedenia humboldti* n. sp. from the South-East Pacific.

Crez

Reconstruction 1

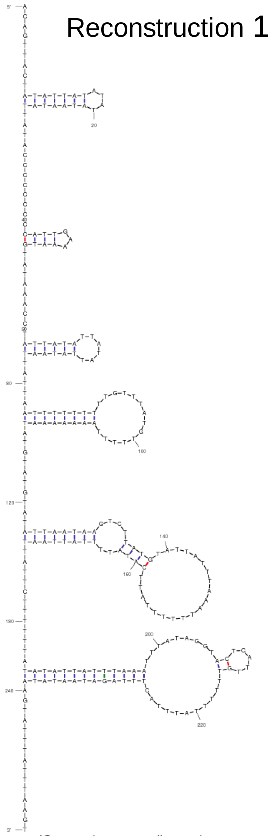

dG = -23.428 non coding region

Reconstruction 2

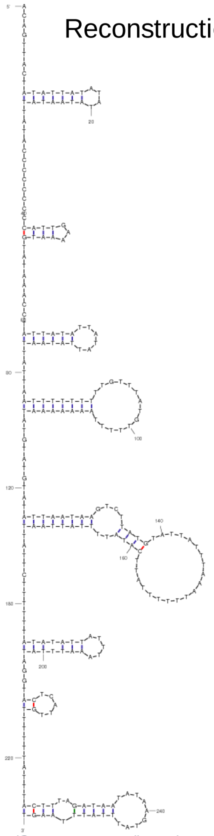

dG = -23.088 non coding region

Reconstruction 3

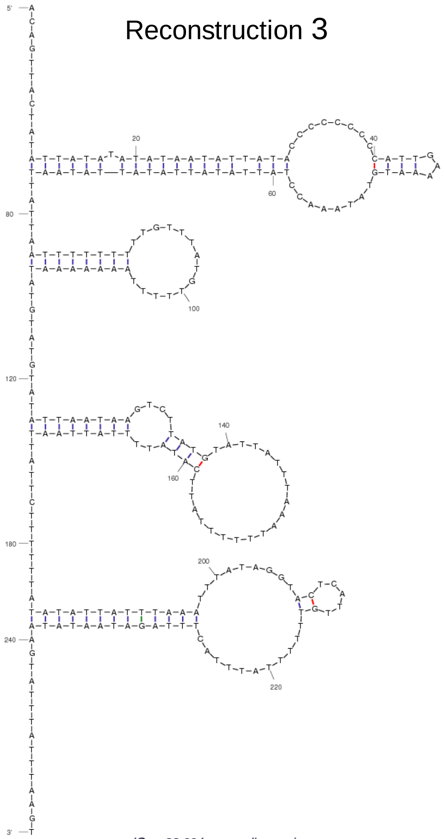

dG = -22.894 non coding region
